# Genomic landscape of endometrial stromal sarcoma of uterus

**DOI:** 10.18632/oncotarget.5384

**Published:** 2015-09-30

**Authors:** Youn Jin Choi, Seung-Hyun Jung, Min Sung Kim, In-Pyo Baek, Jae-Keun Rhee, Sung Hak Lee, Soo Young Hur, Tae-Min Kim, Yeun-Jun Chung, Sug Hyung Lee

**Affiliations:** ^1^ Department of Pathology, College of Medicine, The Catholic University of Korea, Seoul 137-701, Republic of Korea; ^2^ Department of Cancer Evolution Research Center, College of Medicine, The Catholic University of Korea, Seoul 137-701, Republic of Korea; ^3^ Department of Integrated Research Center for Genome Polymorphism, College of Medicine, The Catholic University of Korea, Seoul 137-701, Republic of Korea; ^4^ Department of Medical Informatics, College of Medicine, The Catholic University of Korea, Seoul 137-701, Republic of Korea; ^5^ Department of Hospital Pathology, College of Medicine, The Catholic University of Korea, Seoul 137-701, Republic of Korea; ^6^ Department of Microbiology, College of Medicine, The Catholic University of Korea, Seoul 137-701, Republic of Korea; ^7^ Department of Obstetrics/Gynecology, College of Medicine, The Catholic University of Korea, Seoul 137-701, Republic of Korea

**Keywords:** endometrial stromal sarcoma, mutation, genome, whole exome, copy number

## Abstract

Although recurrent gene fusions such as *JAZF1-JJAZ1* are considered driver events for endometrial stromal sarcoma (ESS) development, other genomic alterations remain largely unknown. In this study, we performed whole-exome sequencing, transcriptome sequencing and copy number profiling for five ESSs (three low-grade ESS (LG-ESS) and two undifferentiated uterine sarcomas (UUSs)). All three LG-ESSs exhibited either one of *JAZF1-SUZ12, JAZF1-PHF1* and *MEAF6-PHF1* fusions, whereas the two UUSs did not. All ESSs except one LG-ESS exhibited copy number alterations (CNAs), many of which encompassed cancer-related genes. In UUSs, five CNAs encompassing cancer-related genes (*EZR, CDH1, RB1, TP53* and *PRKAR1A*) accompanied their expressional changes, suggesting that they might stimulate UUS development. We found 81 non-silent mutations (35 from LG-ESSs and 46 from UUSs) that included 15 putative cancer genes catalogued in cancer-related databases, including *PPARG* and *IRF4* mutations. However, they were non-recurrent and did not include any well-known mutations, indicating that point mutations may not be a major driver for ESS development. Our data show that gene fusions and CNAs are the principal drivers for LG-ESS and USS, respectively, but both may require additional genomic alterations including point mutations. These differences may explain the different biologic behaviors between LG-ESS and UUS. Our findings suggest that ESS development requires point mutations and CNAs as well as the gene fusions.

## INTRODUCTION

Endometrial stromal sarcoma (ESS) is a malignant tumor arising from the stroma of endometrium and accounts for approximately 10% of uterine sarcomas [1]. The current World Health Organization recognizes four categories of the ESS: endometrial stromal nodule (ESN), low-grade endometrial stromal sarcoma (LG-ESS), high-grade endometrial stromal sarcoma (HG-ESS) and undifferentiated uterine sarcoma (UUS) [2]. Both HG-ESS and UUS behave more aggressively than LG-ESS and they are categorized by their molecular features [3]. HG-ESS is defined as an ESS with t(10;17)(q22;p13) rearrangement leading to *YWHAE-FAM22* gene fusion, while UUS is a ‘wastebasket category’ that does not harbor any specific chromosomal translocation [4]. A recurrent gene fusion (*JAZF1-JJAZ1*, t(7;17)(p15;q21)) [5–8] and other less common gene fusions (e.g., *PHF1-JAZF1, PHF1-EPC1, MEAF6-PHF1, ZC3H7-BCOR* and *MBTD1-CXorf67*) have been reported in LG-ESS [9–13]. Although the gene rearrangements are main mechanisms for ESS development, it is possible that somatic mutations might stimulate the development as well. Somatic mutation is a major driving force for the development of most tumors, but there has been no report on somatic mutation status in ESSs at whole-exome or whole-genome level. Also, there are only a few analyses for copy number alterations (CNA) and gene-expression profiling for ESSs [14–16].

To further characterize ESS genomes and extend the knowledge on genetic mechanisms for ESS development, the following questions were investigated in this study: (i) whether LG-ESS, HG-ESS and UUS genomes have driver mutations or CNAs; (ii) whether there are any previously uncharacterized gene fusions in either LG-ESS or HG-ESS or UUS; and (iii) whether there are any differences in expression profiling among LG-ESS, HG-ESS and UUS. For these, we analyzed genomes of LG-ESSs and UUSs (Table 1) by whole-exome sequencing (WES), whole-transcriptome sequencing and microarray- comparative genomic hybridization (a-CGH) in this study.

**Table 1:** Clinical and histologic characteristics of five endometrial stromal sarcomas

	Age	Histopathology grade	Diagnosis	Specimen status	TNM
Case 1	32	Low-grade	LG-ESS	Primary	T1bN0M0
Case 2	34	Low-grade	LG-ESS	Primary	T1bN1M0
Case 3	57	Low-grade	LG-ESS	Metastatic	TxNxM1
Case 4	65	High-grade	UUS	Primary	T2aN0M0
Case 5	57	High-grade	UUS	Metastatic	TxNxM1

LG-ESS: low-grade ESS, UUS: undifferentiated uterine sarcoma, TNM: tumor, lymph node and metastasis

## RESULTS

### Copy number alterations and gene expression profiles

**Figure 1:**
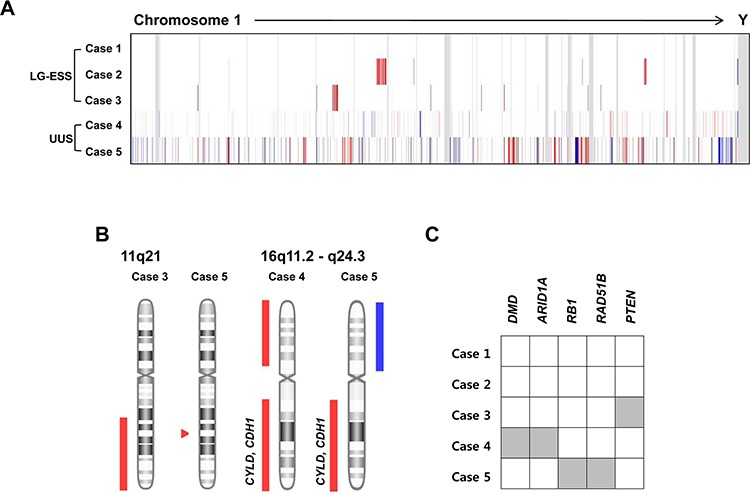
Copy number alteration (CNA) profiles of five endometrial stromal sarcomas **A.** A heatmap for probe-level signal intensities (log_2_ ratios) of five endometrial stromal sarcomas. (Blue: CNA gains, Red: CNA losses) **B.** Recurrent CNAs (*n* ≥ 2) on 11q21 (CNA loss) and 16q11.2 - q24.3. The CNA loss on 16q encompasses candidate tumor suppressor genes *CYLD* and *CDH1*. (Blue: CNA gains, Red: CNA losses) **C.** Five genes (grey) that are catalogued in pan-cancer CNA driver database [19].

A total of 40 CNAs (12 gains and 28 losses) were identified in the five ESSs (LG-ESSs: cases 1–3 and UUSs: cases 4 and 5) by a-CGH (Supplementary Table S1). Of note, one LG-ESS (case 1) did not harbor any CNAs while the other four ESSs harbored CNAs (Figure 1A). To address whether the mutations found in our study could be causally implicated in ESS development, we queried the cancer Gene Census, the CHASM analysis and the pan-cancer databases [17–19]. All four ESS genomes with CNAs harbored one or more CNAs encompassing the cancer-related genes (Table 2). LG-ESSs (cases 2 and 3) harbored CNA losses with the cancer-related genes *PMS2* and *PTEN*, while UUSs (cases 4 and 5) harbored CNA gains of *POU5F1, EZR, GNAQ* and *SYK*, and CNA losses of *ARID1A, DMD, RB1, DICER1, CYLD, CDH1, TP53* and *PRKAR1A*. We also observed recurrent CNAs (*n* ≥ 2) on 11q21 (CNA loss) and 16q11.2 - q24.3 (CNA loss) (Figure 1B). Of note, all the UUSs harbored CNA loss on 16q, where tumor suppressor genes *CYLD* and *CDH1* reside. In addition, we found five genes with CNA losses (*DMD, ARID1A, RB1, RAD51B* and *PTEN*) that overlapped pan-cancer CNA drivers (Figure 1C) [19].

**Table 2:** Summary of gains and losses in five endometrial stromal sarcomas detected by microarray-CGH

	Diagnosis	Gains	Losses	Cancer-related genes
Case 1	LG-ESS	none	none	none
Case 2	LG-ESS	none	7p22.3 - p14.2, 18p11.32 - p11.21	*PMS2*
Case 3	LG-ESS	none	5q22.3 - q34, 10q22.3 - q24.2, 11q21, 15q22.1 - q22.2	*PTEN*
Case 4	UUS	6p21.33 - p21.32, 6q21, 6q24.3 - q27, 8q12.1 - q13.1, 13q14.2 - q14.3, 13q21.2, 14q32.33,	1p36.33 - p35.3, 9p24.1 - p21.3, 9p21.3 - p13.1, 16p13.3 - p11.2, 16q11.2 - q24.3, 19q11 - q13.43, Xp22.33 - p11.21, Xq21.32 - q22.1	*ARID1A, POU5F1, EZR, DMD*, CYLD*, CDH1*
Case 5	UUS	9q21.11 - q31.3, 14q12 - q22.1, 16p13.3 - p11.2, 18p11.32, Xq13.1 - q28	2q36.3 - q37.3, 4q21.1, 4q24 - q25, 4q25, 4q34.1 - q35.2, 6p25.3 - p12.1, 11q14.1 - q25, 13q14.2 - q14.3, 13q21.33, 14q22.1 - q32.33, 16q11.2 - q24.3, 17p13.1, 17q21.33 - q25.3, 22q13.33	*GNAQ, SYK, RB1, RAD51B*, DICER1, CYLD*, CDH1*, TP53, *PRKAR1A*

* The cancer-related genes that were recurrently harbored in CNAs in UUSs (*n* ≥ 2).

LG-ESS: low-grade ESS, UUS: undifferentiated uterine sarcoma

**Figure 2:**
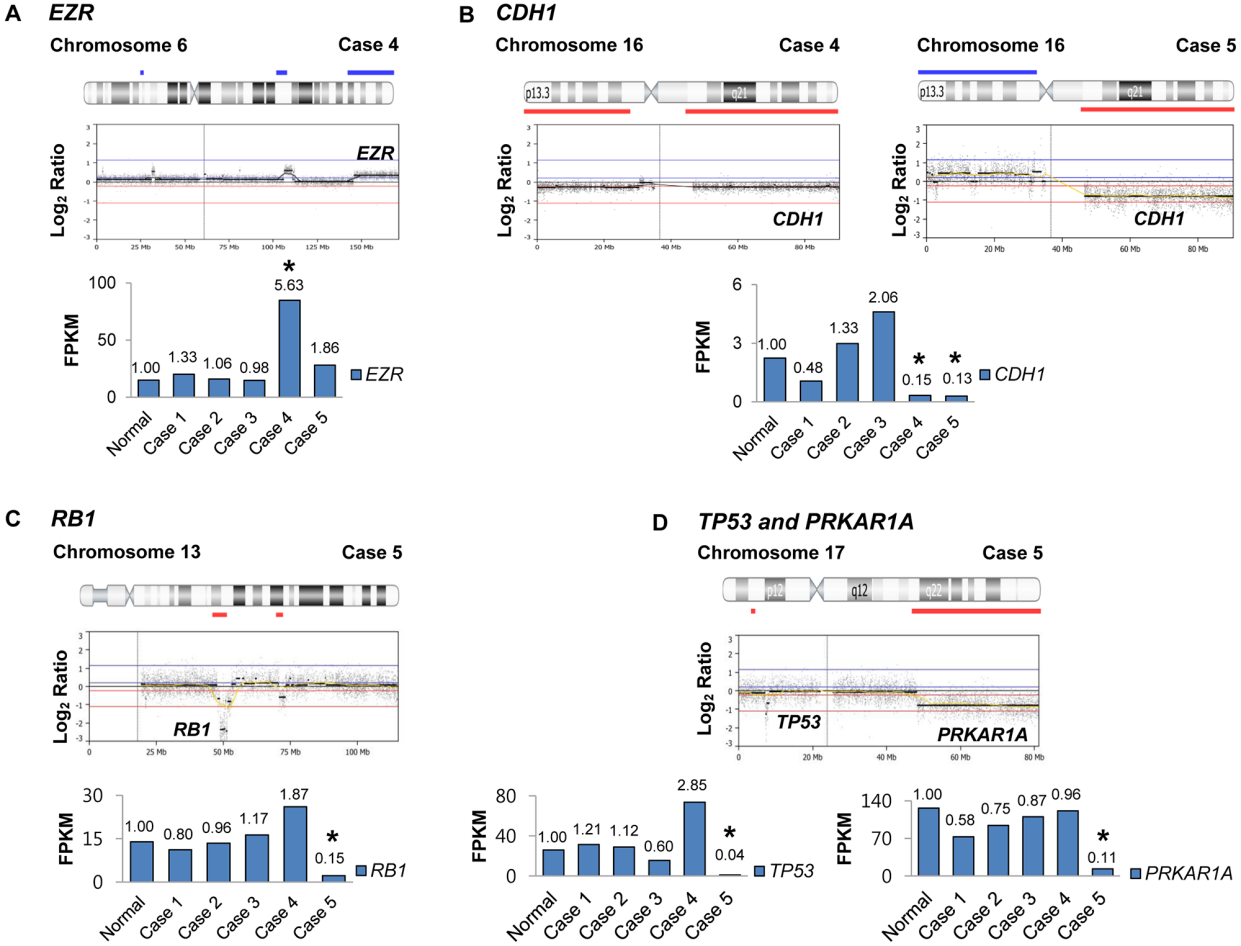
The loci maps of cancer-related genes with copy number alterations and correlated gene expressions Each of the copy number loci is denoted with the corresponding gene. The graph depicts gene expression levels of five endometrial stromal sarcoma tissues as compared to the normal endometrial tissue (fold-changes are shown above the graph). The cases where expressional changes accompany CNAs are marked with asterisk (*). (FPKM: fragments per kilobase of exon per million fragments mapped) **A.** Case 4 harbors a copy number alteration (CNA) gain on *EZR* locus that exhibits expressional increase. **B.** Cases 4 and 5 harbor CNA losses on *CDH1* locus that exhibits expressional decrease. **C.** Case 5 harbors a homozygous CNA loss on *RB1* locus that exhibits expressional decrease. **D.** Case 5 harbors CNA losses on *TP53* and *PRKAR1A* loci that exhibit expressional decreases.

We analyzed gene expression profiles of the five ESSs using the whole-transcription data, which were subsequently compared to their CNA profiles. A normal (non-malignant) endometrial tissue was used to compare the gene expression level with ESS tissues. Five cancer-related genes in the UUSs exhibited a positive correlation between CNAs and gene expressions (down-regulation of genes with CNA loss: *CDH1, RB1, TP53* and *PRKAR1A*; up-regulation of a gene with CNA gain: *EZR*) (Figure 2). Compared to the normal endometrium, the ESSs showed increase of *EZR* expression (5.63 fold-change) and decreases of *RB1* (0.15 fold-change), *CDH1* (0.15 and 0.13 fold-changes in case 4 and case 5, respectively)*, TP53* (0.04 fold-change) and *PRKAR1A* (0.11 fold-change) expressions. All of the copy number losses except *RB1* were heterozygous deletion while *RB1* loss was homozygous deletion (Figure 2).

### Gene fusions

In this study, a total of 536 putative gene fusions (mean, 107; range, 77–150) were identified by whole-transcriptome sequencing (Supplementary Table S2) from the five ESS samples. Of them, we focused on the previously reported genes with translocations based on the Mitelman Database of Chromosome Aberrations (http://cgap.nci.nih.gov/Chromosomes/Mitelman) and found three gene fusions (*JAZF1-SUZ12, JAZF1-PHF1* and *MEAF6-PHF1*). All of them, however, were well-known fusions in ESS. These fusions were detected in the LG-ESSs (cases 1–3), but not in the UUSs (Table 3). The fusions were subsequently validated by reverse transcription (RT)-polymerase chain reaction (PCR) and Sanger sequencing (Supplementary Figure S1).

**Table 3:** Gene fusions detected in endometrial stromal sarcomas

		5′-partner gene					3′-partner gene				
Diagnosis	Sample ID	Gene	Reference sequence	Chromosome	Break position	Strand	Gene	Reference sequence	Chromosome	Break position	Strand
LG-ESS	Case 1	*MEAF6*	ENSG00000163875	1	37967405	+	*PHF1*	ENSG00000112511	6	33380025	-
LG-ESS	Case 2	*JAZF1*	ENSG00000153814	7	27934839	+	*SUZ12*	ENSG00000178691	17	30267305	-
LG-ESS	Case 3	*JAZF1*	ENSG00000153814	7	27934839	+	*PHF1*	ENSG00000112511	6	33380027	-

LG-ESS: low-grade ESS

### Whole-exome sequencing profiles

**Figure 3:**
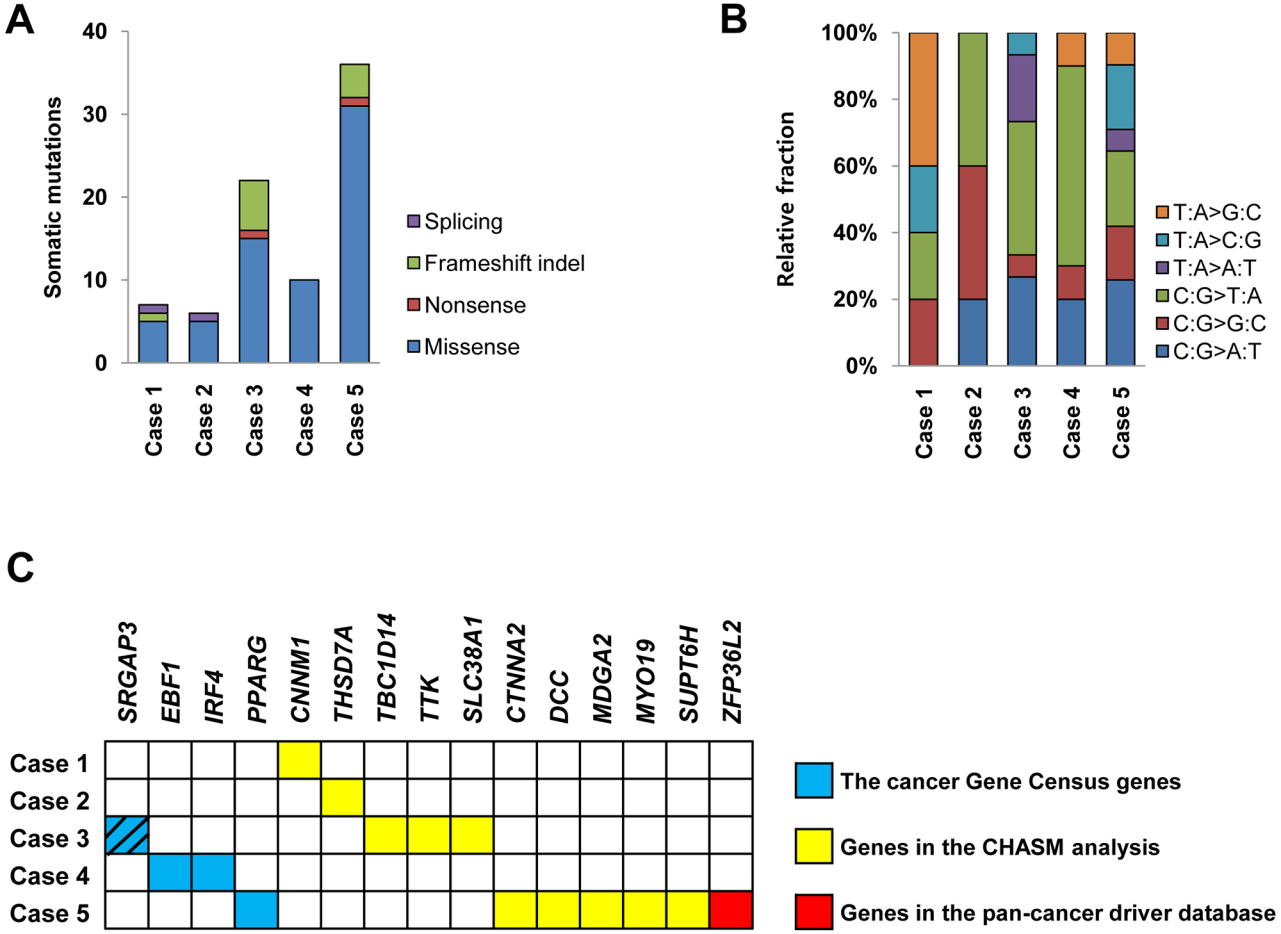
Somatic mutations of five endometrial stromal sarcomas **A.** and **B.** The numbers of somatic mutations and their relative fractions of sequence spectra of five endometrial stromal sarcomas are shown. **C.** Fifteen putative cancer-related genes with somatic mutations. Blue box denotes the genes that overlap the cancer Gene Census genes [17], yellow box denotes the genes that were detected by the CHASM analysis [18] and red box denotes the gene catalogued in the pan-cancer driver database [19]. Of them, *SRGAP3* was detected in both of the cancer Gene Census and the pan-cancer driver database (hatched).

The five ESSs (three LG-ESSs and two UUSs) were also analyzed by WES. Mean coverage of the sequencing depth was 116X (range: 88–143X) for normal samples and 142X (range: 128–155X) for tumor samples. A total of 81 non-silent point mutations and indels (median: 10, range: 6–36) were identified in the five ESSs (Supplementary Table S3). Both distribution of sequence composition and relative fraction of mutation spectra were not significantly different among the five ESSs (*P* > 0.05). Missense mutation with C:G > T:A transition was the most common type (Figures 3A and 3B).

To address whether the mutations found in our study could be causally implicated in ESS development, we queried the cancer Gene Census and found four mutations (*SRGAP3, EBF1, IRF4* and *PPARG*). Also, by analyzing the CHASM to distinguish driver and passenger mutations, we identified ten putative cancer-related mutations (*THSD7A, TBC1D14, TTK, SLC38A1, CTNNA2, SUPT6H, DCC, MDGA2, CNNM1* and *MYO19*) [18]. Finally, in the pan-cancer database, we detected *SRGAP3* and *ZFP36L2* as putative cancer-related genes [19]. Together, we detected 15 putative cancer-related genes with somatic mutations that could be causally implicated in ESS development (Figure 3C). Of them, *SRGAP3* was co-detected in the cancer Gene Census and the pan-cancer database (Figure 3C). Sanger validation of the mutations including four putative cancer-related genes (*SRGAP3, PPARG, DCC* and *ZFP36L2*) is illustrated in Supplementary Figure S2. Of the mutated genes identified, three genes, which included a cancer-related gene *MYO19*, were found to have expressional changes in the same cases by the transcriptome analysis (Supplementary Figure S3).

## DISCUSSION

The aim of this study was twofold. First, we attempted to disclose any somatic genetic alterations other than the gene fusions in ESSs. Second, we for the first time attempted to disclose genetic features of UUSs that by definition do not harbor any of the ESS-specific fusions. We found that the ESSs analyzed in this study harbored 6–36 non-silent somatic mutations per genome, but they did not include well-known mutations (e.g., *TP53, KRAS* and *PIK3CA*). As for the CNAs, all except one ESS harbored CNAs, many of which encompassed well-known driver genes. Importantly, some cancer-related genes in UUSs showed a close correlation between CNAs and gene expression changes, strongly suggesting their implications on ESS tumorigenesis. The UUSs showed bigger median values of CNAs, non-silent mutations and putative cancer-related genes than the LG-ESSs (Table 4), but the differences were not significant, probably due to the small number of the ESSs analyzed. Together, this study identified that ESSs harbored not only ESS-specific fusions but also somatic mutations and CNAs encompassing driver genes. Our findings suggest a possibility that gene fusions alone may not fully develop ESSs as identified in other tumors [20].

**Table 4:** Summary of comparison data between LG-ESS and UUS genomes

	LG-ESS (*n* = 3)	UUS (*n* = 2)	*P* value
Number of CNA	6	34	0.08
Length of CNA	129 Mb	746 Mb	0.08
Cancer-related genes in which copy number and gene expression are correlated	0	5	0.05
Gene fusions	3	0	0.05
Non-silent somatic mutation numbers	35	46	0.25
Putative driver genes (15 somatic mutations and five CNAs)	9	11	0.56

CNA: copy number alteration, LG-ESS: low-grade endometrial stromal sarcoma, UUS: undifferentiated uterine sarcoma

We identified five genes in UUSs that shared CNA and gene expression changes (down-regulation and CNA loss: *PRKAR1A, CDH1, RB1* and *TP53,* up-regulation and CNA gain: *EZR*). *CDH1* is a tumor suppressor gene that encodes E-cadherin and is known to be associated with various malignancies [21, 22]. Loss of *CDH1* results in dysfunction of cell-cell adhesion, allowing for abnormal cell-cell interaction such as epithelial-mesenchymal transition (EMT) [23]. In addition, *CDH1* behaves as a negative regulator in Wnt signaling [24]. ESSs are related to up-regulation of Wnt signaling, for example, exhibiting a down-regulation of *SFRP4*, a negative regulator in Wnt signaling [22, 25]. In our study, we also found that both *SFRP4* and *CDH1* expressions were decreased in the ESSs, further suggesting the importance of Wnt signaling in ESSs. Recent research advances yielded a number of US Food and Drug Administration (FDA)-approved drugs that may change Wnt signaling [26]. Our study may provide further rationale for performing future studies that may explore the use of Wnt modulators in ESSs. *TP53* is the most common tumor suppressor gene that is frequently inactivated in most cancers [27]. Tumor suppressor genes generally follow “two-hit hypothesis”, being bi-allelically inactivated by point mutation, deletion and promoter hypermethylation [28]. In spite of the earlier report on positive *TP53* mutations in ESSs [29], we did not identify any *TP53* point mutations in the ESSs by WES. In a UUS (case 5), *TP53* locus was deleted heterozygously and *TP53* expression was decreased. Together, these data indicate that *TP53* inactivation in ESSs may result from point mutation or deletion and suggest a possibility that haploinsufficiency of *TP53* might inactivate its tumor suppressor roles. Similarly, chronic lymphocytic leukemia exhibited both bi-allelic and mono-allelic *TP53* gene [30]. By contrast, we observed that another tumor suppressor gene *RB1* exhibited homozygous deletion in another UUS (case 5).

Recurrent fusion transcripts are frequently detected in ESSs [4, 5, 9–13]. *JAZF1-SUZ12, JAZF1-PHF1* and *MEAF6-PHF* are gene-fusions that have been detected most frequently in LG-ESSs [5, 12, 13] and *YWHAE-FAM22* gene fusion is a key factor in defining HG-ESS [31]. By definition, UUSs are high-grade ESSs without known gene fusions [2]. As expected, we were able to find *JAZF1-SUZ12, JAZF1-PHF1* and *MEAF6-PHF* fusions in each of the three LG-ESSs. For the two ESSs without any known ESS-specific fusions, we were not able to detect any novel fusions, identifying that they were UUSs per se. Our data suggest that the UUS with a driver fusion might, if any, be very rare.

We found *PPARG* and *IRF4* mutations in the ESSs. *PPARG* encodes peroxisome proliferator-activated receptor gamma (PPAR-γ/PPARG) [32] that has tumor suppressor functions in many endocrine organs including breast, prostate and pituitary gland [33–35]. In uterus, PPARG activation inhibits growth and survival of human endometriosis cells by suppressing estrogen biosynthesis [36]. *IRF4* encodes a transcription factor in interferon regulatory factor family. A chromosomal translocation involving *IRF4* and the IgH locus, t(6;14)(p25;q32) is considered a cause of multiple myeloma [37]. IRF4 is required for endometrial decidualization [38]. Together, these data suggest a possible rationale that *PPARG* and *IRF4* mutations might be involved in ESS development.

**Figure 4:**
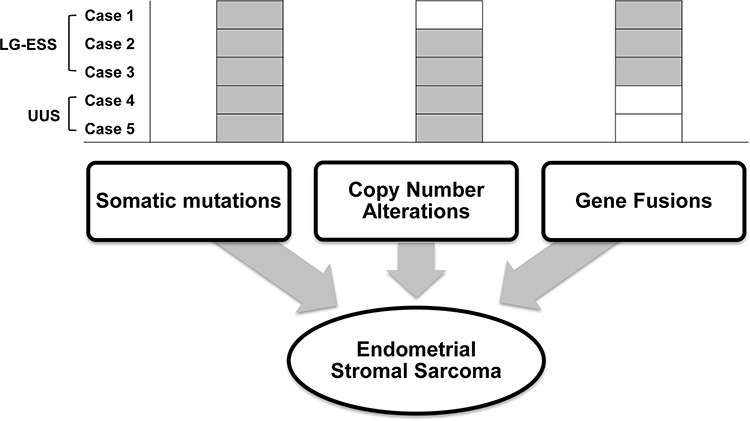
Schematic representation of suggested genetic alterations in endometrial stromal sarcoma Somatic mutations and copy number alterations as well as gene fusions are found in the endometrial stromal sarcomas. Compositions of these genomic alterations are summarized for each ESS.

In this study, ESSs harbored at least one genetic alteration (somatic mutations or CNAs or gene fusions) that may stimulate ESS tumorigenesis (Figure 4). Also, we observed that all of the ESSs carried either somatic mutations or CNAs-harboring driver genes, albeit not recurrent. It suggests that non-recurrent alterations may cooperate together for the ESS turmorigenesis.

Our data are based on the analysis of five ESS genome pairs (three LG-ESSs and two UUSs). The small sample size is due to the rarity of ESS [1, 2]. Further investigation with larger sample size across diverse ethnic groups would reveal additional information, e.g., discovery of potential additional driver mutations in ESSs and additional novel fusions in LG-ESSs and HG-ESSs. In addition, a larger cohort would provide genomic features of metastatic ESSs compared to the primary ESSs.

In summary, our data for the first time attempted the integrative analyses of whole-exome, whole-transcriptome and a-CGH in ESS genomes. Previous studies on ESS genomes mainly focused on gene fusions. However, our data indicate that fusions are not the only genetic alteration occurred during ESS development. Somatic mutations, CNAs as well as gene fusions alone or together might contribute to ESS development. Our data also suggest that CNAs may be a major genomic alteration for UUS development while gene fusions are the major genomic alteration for LG-ESS. Our findings may provide a useful resource for understanding this heterogeneous disease and identifying genomic clues for differential diagnosis and therapy options for ESS.

## MATERIAL AND METHODS

### Endometrial stromal sarcoma tissues

Normal and tumor tissues from five ESS patients were obtained from the tissue banks of Korean Gynecologic Cancer Bank (Seoul, Korea), Guro Hospital of Korea University (Seoul, Korea) and Busan National University Hospital (Busan, Korea). We also used normal endometrial tissue from a healthy woman. All of the six samples were from Koreans. Approval for this study was obtained from the institutional review board at the Catholic University of Korea, College of Medicine. Clinicopathologic features of the five ESS patients are summarized in Table 1. Initially, frozen tissues from the tissue banks were cut, stained with the hematoxylin/eosin and examined under microscope by a pathologist, who identified areas rich in ESS tumor cells in the frozen tissues. In order to procure matched normal tissues from each ESS patient, we used peripheral blood lymphocytes or normal tissues that were confirmed to be free of tumor cells by microscopic examination. Purities of the tumor cells were approximately 70%. For genomic DNA and RNA extraction from the frozen tissues, we used the DNeasy Blood & Tissue Kit (Qiagen, Hilden, Germany) and mirVanamiRNA Isolation Kit (Invitrogen, Carlsbad, CA, USA), respectively.

### Whole-exome sequencing

Using genomic DNA from five ESSs and matched normal cells, we performed exome-capture using the Agilent SureSelect Human All Exome 50Mb kit (Agilent Technologies) according to the manufacturer's instructions. DNA libraries were constructed according to the protocol provided by the manufacturer and whole exome-sequencing (WES) was performed using an Illumina HiSeq2000 platform to generate 101bp paired-end reads. Burrows-Wheeler aligner was used to align the sequencing reads onto the human reference genome (hg19). The aligned sequencing reads were evaluated by using Qaulimap [39]. Supplementary Table S4 shows the information of sequencing alignments (e.g., the number of reads and sequencing coverage). Processing and the management of the sequencing data were performed as described elsewhere [20]. In brief, somatic variants were identified by using MuTect [40] and SomaticIndelDetector [41] for point mutations and indels. ANNOVAR package was used to select somatic variants located in coding sequences and to predict their functional consequences, such as silent or non-silent variants [42]. Then, we used the CHASM analysis program with ‘uterus’ option for cancer tissue type (FDR < 0.3) in order to identify the putative cancer-related mutations [18]. In order to validate noticeable somatic mutations, genomic DNA from tumor areas and matched normal tissues from each case were amplified by PCR and sequence analyses were performed.

### Transcriptome sequencing for gene fusion and expression profiling analyses

The mRNA of five ESSs and normal proliferative endometrium of a woman was converted into a 272bp to 289bp-sized cDNA library using TruSeq RNA Library Preparation Kit (Illumina). Whole-transcriptome sequencing was performed using an Illumina HiSeq2000 platform. Sequencing reads were mapped onto the human reference genome (GRCh37, hg19). Gene fusions were identified by searching for the spanning reads and split reads by using the deFuse program [43]. Transcriptome sequencing data were analyzed using TopHat (http://ccb.jhu.edu/software/tophat/index.shtml) for alignment, Cufflinks for assembly [44] and a known set of reference transcripts from Ensembl v. 65 (http://www.ensembl.org) for expression estimation. The expression levels were quantified as fragments per kilobase of exon per million fragments mapped (FPKM).

### Reverse transcription-polymerase chain reaction and sequencing

RT was performed using oligo-(dT) primer and SuperScript III reverse transcriptase (Invitrogen). PCR was performed with Pfu DNA polymerase (Promega) according to the manufacturer's instruction. The thermal cycling included one cycle at 95°C for 2 min followed by 35 cycles of 95°C at 0.5 min, 55–61°C for 0.5 min, 72°C for 1 min and a final extension of 72°C for 5 min. Details of the primer pairs and corresponding genes are available in Supplementary Table S5. PCR products were visualized on 2% agarose gel and subsequently analyzed by direct DNA sequencing.

### DNA copy number profiling

DNA copy number profiling was performed using the Agilent Sure Print G3 Human CGH Microarray 180K. The genomic DNA of five ESS tissues and matched normal tissues was hybridized onto the array according to the manufacturer's instructions. Background correction and normalization for array images was performed using Agilent Feature Extraction Software v10.7.3.1. The RankSegmentation statistical algorithm in NEXUS software v7.5 (Biodiscovery Inc., El Segundo, CA) was used to define the CNAs of each sample; a log2 ratio larger than 0.3 was identified as gain and lower than −0.3 as loss.

## SUPPLEMENTARY FIGURES AND TABLES






