# Supplementary material for: Genomic landscape of endometrial stromal sarcoma of uterus
Source: Oncotarget. 2015 Sep 30;6(32):33319–28. doi: 10.18632/oncotarget.5384 (PMC4741768; doi:10.18632/oncotarget.5384)
Supplement: Supplementary file 1 [file oncotarget-06-33319-s001.pdf]

SUPPLEMENTARY FIGURES AND TABLES

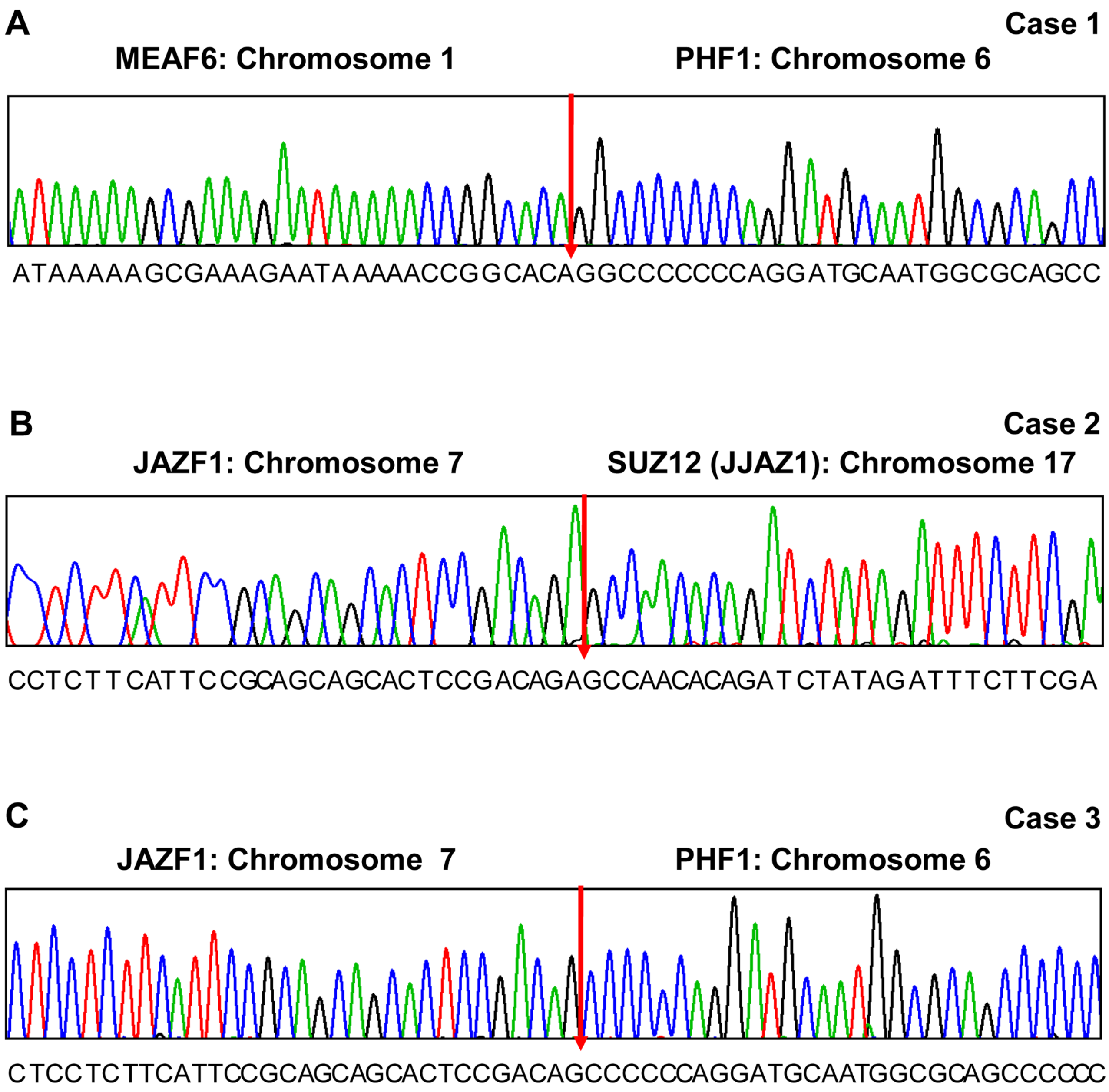

Supplementary Figure S1: Gene fusions identified in the five endometrial stromal sarcomas. Three known gene fusions were detected in low-grade endometrial stromal sarcomas; **A.** *MEAF6-PHF1*, **B.** *JAZF1-SUZ12* (*JJAZ1*) and **C.** *JAZF1-PHF1*.

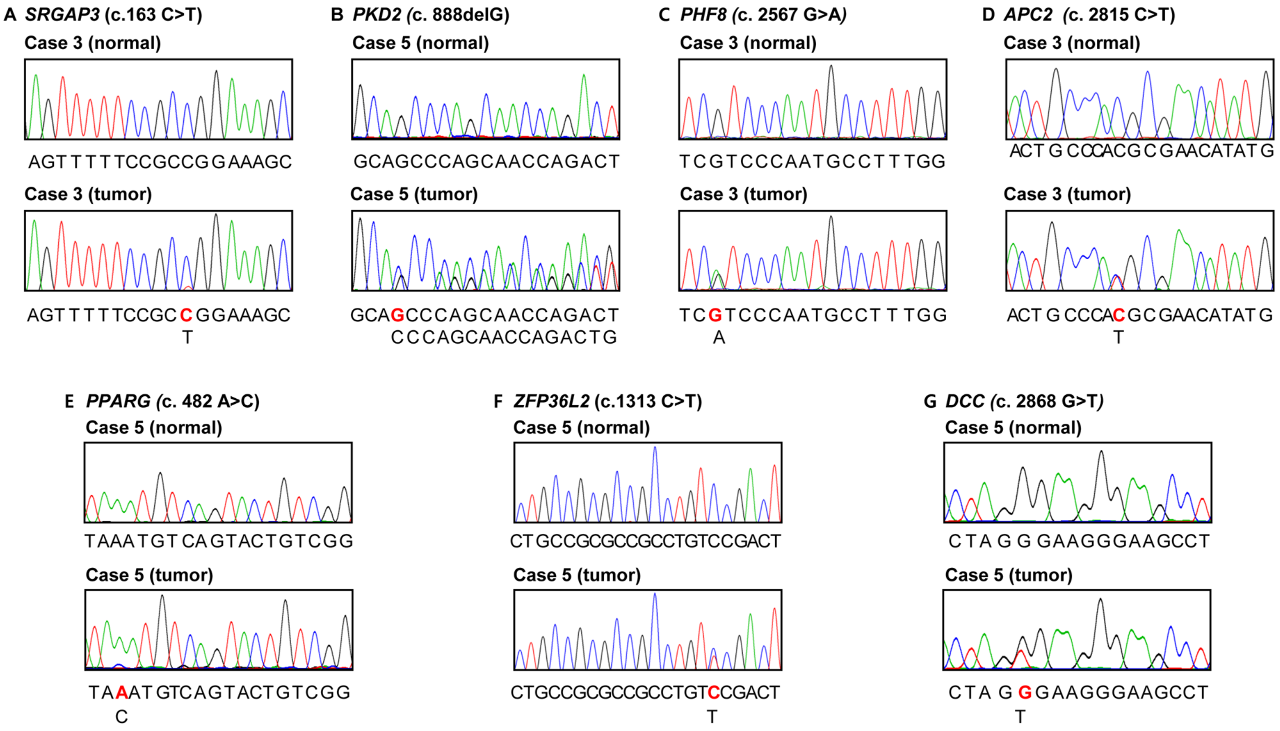

Supplementary Figure S2: Validation of the mutations including four putative cancer-related genes (*SRGAP3, PPARG, DCC* and *ZFP36L2*) by Sanger sequencing.

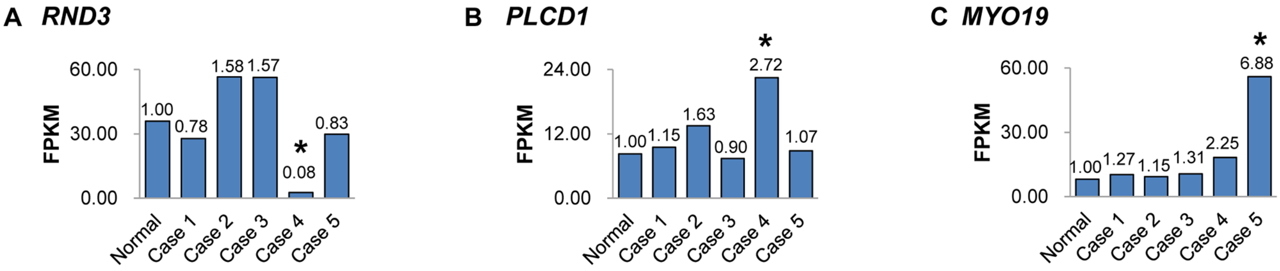

Supplementary Figure S3: Co-occurrence of somatic point mutations and expressional changes. The graph depicts gene expression levels with indicated genes (**A.** *RND3*, **B.** *PLCD1*, **C.** *MYO19*) as compared to the expression in normal endometrial tissue (fold-changes are shown above the graph). The cases where expressional changes and somatic point mutations are marked with asterisk (*). (FPKM: fragments per kilobase of exon per million fragments mapped)

Supplementary Table S1: Copy number alterations of five endometrial stromal sarcoma tissues by array-comparative genomic hybridization

Sample ID	Chromosome	Start	End	Event	Length	Cytoband	Probe Median	The cancer Gene Census genes
Case 2	7	0	36142309	CN Loss	36142309	p22.3 - p14.2	−0.795	CARD11, PMS2, ETV1, HNRNPA2B1, HOXA9, HOXA11, HOXA13, JAZF1
Case 2	18	0	14966054	CN Loss	14966055	p11.32 - p11.21	−0.779	
Case 3	5	113680564	168394895	CN Loss	54714332	q22.3 - q34	−0.866	PDGFRB, CD74, ITK, EBF1
Case 3	10	81665814	100179075	CN Loss	18513262	q22.3 - q24.2	−0.858	BMPR1A, FAM22A, PTEN
Case 3	11	94462357	96080802	CN Loss	1618446	q21	−0.906	MAML2
Case 3	15	59213300	62421973	CN Loss	3208674	q22.1 - q22.2	−0.861	
Case 4	1	1104109	28564365	CN Loss	27460257	p36.33 - p35.3	−0.216	TNFRSF14, PRDM16, RPL22, CAMTA1, SDHB, PAX7, MDS2, ARID1A
Case 4	6	31124853	32965567	CN Gain	1840715	p21.33 - p21.32	0.53	POU5F1
Case 4	6	106075906	112225218	CN Gain	6149313	q21	0.544	PRDM1
Case 4	6	145653505	171115067	CN Gain	25461563	q24.3 - q27	0.344	EZR, FGFR1OP, MLLT4
Case 4	8	59785804	67046677	CN Gain	7260874	q12.1 - q13.1	0.597	
Case 4	9	5484285	21962671	CN Loss	16478387	p24.1 - p21.3	−0.332	NFIB, MLLT3
Case 4	9	22008411	39156954	CN Loss	17148544	p21.3 - p13.1	−0.318	FANCG, PAX5
Case 4	13	48880498	54377925	CN Gain	5497428	q14.2 - q14.3	0.599	RB1
Case 4	13	60452783	60821443	CN Gain	368661	q21.2	0.592	
Case 4	14	106255536	106722310	CN Gain	466775	q32.33	0.517	
Case 4	16	1	30535371	CN Loss	30535371	p13.3 - p11.2	−0.275	TSC2, CREBBP, CIITA, SOCS1, TNFRSF17, ERCC4, MYH11, PALB2, IL21R

(*Continued*)

Sample ID	Chromosome	Start	End	Event	Length	Cytoband	Probe Median	The cancer Gene Census genes
Case 4	16	46500741	90354753	CN Loss	43854013	q11.2 - q24.3	−0.264	CYLD, HERPUD1, CDH11, CBFB, CDH1, MAF, CBFA2T3, FANCA
Case 4	19	28419592	59128983	CN Loss	30709392	q11 - q13.43	−0.204	CCNE1, CEBPA, AKT2, CD79A, CIC, BCL3, CBLC, ERCC2, KLK2, PPP2R1A, ZNF331, TFPT
Case 4	X	1	56992543	CN Loss	56992543	p22.33 - p11.21	−0.27	CRLF2, P2RY8, ZRSR2, BCOR, KDM6A, SSX1, SSX4, WAS, GATA1, TFE3, SSX2, KDM5C
Case 4	X	92526140	99600043	CN Loss	7073904	q21.32 - q22.1	−0.281	
Case 5	2	229977278	243199373	CN Loss	13222096	q36.3 - q37.3	−0.821	
Case 5	4	77348395	78320138	CN Loss	971744	q21.1	−0.675	
Case 5	4	107377307	108806025	CN Loss	1428719	q24 - q25	−0.579	
Case 5	4	111487353	112458961	CN Loss	971609	q25	−0.548	
Case 5	4	173890887	191154276	CN Loss	17263390	q34.1 - q35.2	−0.686	
Case 5	6	0	53532579	CN Loss	53532580	p25.3 - p12.1	−0.672	IRF4, DEK, HIST1H4I, TRIM27, POU5F1, DAXX, HMGA1, FANCE, PIM1, TFEB, CCND3
Case 5	9	70984481	114823657	CN Gain	43839177	q21.11 - q31.3	0.51	GNAQ, SYK, OMD, FANCC, XPA, NR4A3, TAL2
Case 5	11	83521585	135006516	CN Loss	51484932	q14.1 - q25	−0.72	PICALM, MAML2, BIRC3, ATM, DDX10, POU2AF1, SDHD, PAFAH1B2, PCSK7, MLL, DDX6, CBL, ARHGEF12, FLI1

(*Continued*)

Sample ID	Chromosome	Start	End	Event	Length	Cytoband	Probe Median	The cancer Gene Census genes
Case 5	13	47808341	53026790	CN Loss	5218450	q14.2 - q14.3	−1.443	RB1
Case 5	13	70811820	73082806	CN Loss	2270987	q21.33	−0.588	
Case 5	14	26677716	51437992	CN Gain	24760277	q12 - q22.1	0.901	NKX2-1, NIN
Case 5	14	51437992	107349540	CN Loss	55911549	q22.1 - q32.33	−0.738	KTN1, GPHN, TSHR, TRIP11, GOLGA5, DICER1, TCL6, TCL1A, BCL11B, AKT1
Case 5	16	0	33831749	CN Gain	33831750	p13.3 - p11.2	0.352	TSC2, CREBBP, CIITA, SOCS1, TNFRSF17, ERCC4, MYH11, PALB2, IL21R, FUS
Case 5	16	46500741	90354753	CN Loss	43854013	q11.2 - q24.3	−0.794	CYLD, HERPUD1, CDH11, CBFB, CDH1, MAF, CBFA2T3, FANCA
Case 5	17	7208897	8223551	CN Loss	1014655	p13.1	−0.867	TP53, PER1
Case 5	17	48238730	81195210	CN Loss	32956481	q21.33 - q25.3	−0.773	COL1A1, HLF, MSI2, CLTC, BRIP1, CD79B, DDX5, PRKAR1A, SRSF2, CANT1, ASPSCR1
Case 5	18	0	2403253	CN Gain	2403254	p11.32	0.983	
Case 5	22	50645269	51304566	CN Loss	659298	q13.33	−1.029	
Case 5	X	70528876	155270560	CN Gain	84741685	q13.1 - q28	0.564	ATRX, SEPT6, ELF4, GPC3, PHF6, MTCP1

Supplementary Table S2: Transcriptome data analysis of five endometrial stromal sarcoma tissues.

Supplementary Table S3: Somatic point mutations and indels identified across five endometrial stromal sarcoma genomes.

Supplementary Table S4: The description of whole-exome sequencing data

Sample ID*	Sequencing reads	Mapped (%)	Coverage (mean)	% Coverage of target regions (more than 10X)**
Case 1N	127035946	126309688 (99.4%)	132	0.96
Case 1T	138365442	137941974 (99.7%)	155	0.98
Case 2N	109847080	109243792 (99.5%)	125	0.98
Case 2T	112844424	112429416 (99.6%)	128	0.98
Case 3N	93242160	88593930 (95.0%)	91	0.98
Case 3T	127960674	127413790 (99.6%)	145	0.98
Case 4N	129177898	128700168 (99.6%)	143	0.98
Case 4T	128367250	127804956 (99.6%)	138	0.98
Case 5N	93703090	91133396 (97.3%)	88	0.98
Case 5T	127093702	126428940 (99.5%)	143	0.98

*The neoplasia and matched normal genomes are discriminated with the use of 'T' and 'N', respectively.
**The mean coverage and the % of bases were calculated onto the targeted regions (Agilent SureSelect 50Mb exon).

Supplementary Table S5: Primers used in the RT-PCR assays for validation of gene fusions

Gene fusion	Primer	Sequencing 5′-> 3′
JAZF1-SUZ12 (JJAZ1)	JAZF1-F	CCACAGCAGTGGAAGCCTTA
	SUZ12(JJAZF1)-R	CCGGGTTTTGTTTGATTGAGG
JAZF1-PHF1	JAZF1-F	GCAGCCAACCTATGTTGCCCTGAG
	PHF1-R	AGCCCATCAGTCCATCTGGCCAG
MEAF6-PHF1	MEAF6-F	CATTGGCAGGAGTTCAGGACCAGC
	PHF1-R	GGACCAGACACACCTCCCTAGCACTG
