# Supplementary material for: Genomic landscape of endometrial stromal sarcoma of uterus
Source: Oncotarget. 2015 Sep 30;6(32):33319–28. doi: 10.18632/oncotarget.5384 (PMC4741768; doi:10.18632/oncotarget.5384)
Supplement: Supplementary file 2 [file oncotarget-06-33319-s002.docx]

**Table S2. Transcriptome data analysis of five endometrial stromal sarcoma tissues**

| **Sample_ID** | **gene_chromosome1** | **gene_chromosome2** | **gene_location1** | **gene_location2** | **gene_name1** | **gene_name2** |
| --- | --- | --- | --- | --- | --- | --- |
| Case 1 | 6 | 6 | intron | downstream | LINC00340 | RP11-524C21.1 |
| Case 1 | 14 | 14 | utr5p | intron | RABGGTA | DHRS1 |
| Case 1 | 10 | 10 | coding | intron | DLG5 | RP11-126H7.3 |
| Case 1 | 9 | 9 | intron | intron | RP11-473E2.2 | RP11-473E2.4 |
| Case 1 | 19 | 19 | coding | intron | DKKL1 | TEAD2 |
| Case 1 | 10 | 10 | coding | intron | CRTAC1 | GOLGA7B |
| Case 1 | 7 | 7 | coding | coding | TRBJ2-3 | TRBC2 |
| Case 1 | 11 | 11 | intron | upstream | BCAS2P1 | PCF11 |
| Case 1 | 5 | 5 | intron | downstream | FAM153C | RP11-889L3.1 |
| Case 1 | 6 | 1 | intron | intron | MDN1 | GAS5 |
| Case 1 | 11 | 11 | intron | coding | ARHGAP42 | PGR |
| Case 1 | 13 | 13 | coding | intron | CLDN10 | DZIP1 |
| Case 1 | 7 | 7 | coding | coding | TRBJ2-3 | TRBC2 |
| Case 1 | 9 | 9 | coding | intron | XPA | NCBP1 |
| Case 1 | 19 | 19 | coding | coding | CLDND2 | ETFB |
| Case 1 | 2 | 1 | intron | intron | AC116050.1 | RP11-14N7.2 |
| Case 1 | 11 | 11 | coding | utr5p | SIDT2 | TAGLN |
| Case 1 | X | X | coding | upstream | KDM5C | RP11-258C19.4 |
| Case 1 | 22 | 22 | intron | downstream | SREBF2 | SHISA8 |
| Case 1 | 22 | 22 | coding | downstream | TTLL12 | TTLL12 |
| Case 1 | 7 | 7 | coding | coding | TRBJ2-4 | TRBC2 |
| Case 1 | 7 | 7 | coding | coding | TRBJ2-5 | TRBC2 |
| Case 1 | 1 | 1 | coding | intron | MRPL37 | RP5-997D24.5 |
| Case 1 | 19 | 19 | coding | coding | TGFB1 | HNRNPUL1 |
| Case 1 | 3 | 3 | intron | upstream | GATA2 | TMED10P2 |
| Case 1 | 9 | 4 | intron | coding | PGM5P2 | FRG1 |
| Case 1 | 21 | 21 | coding | intron | YBEY | C21orf58 |
| Case 1 | X | 14 | coding | intron | PPP1R3F | NUBPL |
| Case 1 | 17 | 6 | intron | intron | ZNF652 | SNHG5 |
| Case 1 | 19 | 19 | utr3p | upstream | AC010642.1 | ZSCAN22 |
| Case 1 | 7 | 3 | intron | intron | AC005682.6 | MAP4 |
| Case 1 | 3 | 3 | upstream | intron | MIR1324 | RP11-413E6.7 |
| Case 1 | 14 | 14 | utr5p | coding | RABGGTA | DHRS1 |
| Case 1 | 2 | 1 | intron | intron | AC116050.1 | RP11-14N7.2 |
| Case 1 | 6 | 9 | intron | intron | MTO1 | EEF1A1P5 |
| Case 1 | 2 | 2 | coding | intron | ADCY3 | PTRHD1 |
| Case 1 | 19 | 19 | utr3p | upstream | AC010642.1 | ZSCAN22 |
| Case 1 | 13 | 13 | intron | downstream | FNDC3A | MLNR |
| Case 1 | 9 | 9 | intron | coding | NAA35 | GOLM1 |
| Case 1 | 4 | 2 | coding | coding | CFI | THADA |
| Case 1 | 16 | 16 | intron | coding | Z97634.5 | NME4 |
| Case 1 | 1 | X | coding | coding | CTBS | GNG5P2 |
| Case 1 | 15 | 15 | utr3p | utr3p | ANKDD1A | SPG21 |
| Case 1 | 19 | 19 | utr3p | upstream | NDUFA7 | CD320 |
| Case 1 | 19 | 2 | downstream | intron | RNA5-8SP4 | PID1 |
| Case 1 | 3 | 10 | intron | intron | RP11-413E6.1 | KIAA0664P5 |
| Case 1 | 10 | 10 | upstream | intron | RNA5SP309 | ZEB1 |
| Case 1 | 17 | 6 | intron | intron | ZNF652 | SNHG5 |
| Case 1 | 1 | 1 | coding | intron | PDE4DIP | LINC00623 |
| Case 1 | 19 | 19 | utr3p | coding | IRGQ | PINLYP |
| Case 1 | 3 | 3 | utr5p | downstream | ALG1L | ROPN1B |
| Case 1 | 9 | 9 | downstream | intron | RP11-187C18.6 | RP11-154P18.2 |
| Case 1 | 5 | 5 | utr3p | downstream | ATOX1 | CTB-113P19.1 |
| Case 1 | 3 | 22 | intron | intron | RP11-280F2.2 | TPTEP1 |
| Case 1 | 19 | 19 | coding | downstream | SLC1A5 | FKRP |
| Case 1 | 10 | 10 | intron | intron | RP11-773D16.1 | RP11-506M13.3 |
| Case 1 | 2 | 2 | coding | upstream | AC007405.7 | Y_RNA |
| Case 1 | 4 | 16 | coding | intron | FRG1 | RP11-296I10.6 |
| Case 1 | 2 | 2 | utr3p | intron | SUPT7L | GPN1 |
| Case 1 | 2 | 2 | coding | intron | ACYP2 | AC008280.5 |
| Case 1 | 15 | 1 | intron | intron | GABRB3 | GAS5 |
| Case 1 | 11 | 2 | coding | intron | LSP1 | AC116050.1 |
| Case 1 | 6 | 1 | intron | intron | MDN1 | GAS5 |
| Case 1 | 9 | 9 | utr3p | downstream | KLHL9 | IFNA20P |
| Case 1 | 19 | 9 | intron | upstream | CTD-3162L10.1 | RP11-216M21.1 |
| Case 1 | 19 | 19 | coding | upstream | ZNF544 | ZNF8 |
| Case 1 | 10 | 10 | upstream | intron | RNA5SP309 | ZEB1 |
| Case 1 | 1 | X | coding | coding | CTBS | GNG5P2 |
| Case 1 | 19 | 1 | utr3p | coding | CRX | AL162431.1 |
| Case 1 | 9 | 9 | coding | intron | HIATL1 | RP11-330M2.7 |
| Case 1 | 14 | 9 | utr3p | intron | PACS2 | RP11-65J3.1 |
| Case 1 | 19 | 19 | coding | upstream | TGFB1 | CCDC97 |
| Case 1 | 15 | 1 | intron | intron | GABRB3 | GAS5 |
| Case 1 | X | X | downstream | downstream | TAZ | BX936347.1 |
| Case 1 | 11 | 19 | utr3p | coding | C11orf92 | FBXL12 |
| Case 1 | 2 | 2 | utr3p | intron | CFLAR | AC007283.5 |
| Case 1 | X | X | coding | downstream | RPL39 | SOWAHD |
| Case 1 | 1 | 1 | downstream | intron | RP5-924G13.2 | GAS5 |
| Case 1 | 19 | 19 | upstream | upstream | DUS3L | NRTN |
| Case 1 | 10 | 10 | intron | intron | RP11-773D16.1 | RP11-119F19.2 |
| Case 1 | 2 | 2 | coding | intron | ANAPC1 | AC125232.1 |
| Case 1 | 7 | 9 | intron | intron | AC074183.4 | SNHG7 |
| Case 1 | 2 | 2 | intron | coding | AC008280.5 | ACYP2 |
| Case 1 | 20 | 20 | utr3p | utr3p | SULF2 | NCOA3 |
| Case 1 | 1 | 1 | downstream | intron | RP5-924G13.2 | GAS5 |
| Case 1 | 6 | 6 | utr5p | intron | SLC22A16 | CTA-331P3.1 |
| Case 1 | 14 | 14 | upstream | intron | WDR89 | U3 |
| Case 1 | 9 | 2 | upstream | intron | AL161626.1 | PKP4 |
| Case 1 | 4 | 4 | coding | intron | TMEM165 | CLOCK |
| Case 1 | 7 | 7 | intron | intron | PMS2P3 | TYW1 |
| Case 1 | 10 | 10 | downstream | intron | RP11-564C4.7 | RP11-119F19.2 |
| Case 1 | 12 | 12 | intron | utr3p | DNM1L | YARS2 |
| Case 1 | 11 | 11 | intron | intron | NLRP6 | RP11-326C3.2 |
| Case 1 | 9 | 9 | intron | downstream | RP11-262H14.3 | RP11-211N8.2 |
| Case 1 | 4 | 19 | intron | downstream | AC093323.3 | ANGPTL4 |
| Case 1 | X | 8 | intron | intron | MAGED1 | KB-1471A8.1 |
| Case 1 | 19 | 19 | coding | downstream | CFD | MED16 |
| Case 1 | 7 | 7 | downstream | downstream | RP4-800G7.1 | AC104843.3 |
| Case 1 | X | X | downstream | downstream | TAZ | BX936347.1 |
| Case 1 | 1 | 1 | downstream | intron | RP5-924G13.2 | GAS5 |
| Case 1 | 16 | 16 | utr3p | upstream | NOMO3 | hsa-mir-3179-3 |
| Case 1 | 4 | 13 | intron | upstream | OTOP1 | RPL34P27 |
| Case 1 | 19 | 19 | coding | intron | ZNF814 | ZNF587 |
| Case 1 | 5 | 5 | intron | intron | RP11-141O11.2 | CTC-498J12.1 |
| Case 1 | 4 | 1 | intron | intron | PPEF2 | HFM1 |
| Case 1 | 6 | 1 | utr3p | utr3p | TAF8 | EPHA10 |
| Case 1 | 22 | 7 | utr3p | downstream | LDOC1L | AC093627.9 |
| Case 1 | 11 | 3 | intron | intron | RNH1 | AC069513.3 |
| Case 1 | 10 | 10 | coding | downstream | MSRB2 | YWHAZP3 |
| Case 1 | 15 | 1 | intron | intron | GABRB3 | GAS5 |
| Case 1 | 4 | 4 | coding | upstream | CFI | PLA2G12A |
| Case 1 | 13 | 13 | coding | intron | ABCC4 | RNY3P8 |
| Case 1 | 7 | 7 | upstream | intron | TWIST1 | AC003986.7 |
| Case 1 | 19 | 19 | coding | upstream | KCNK6 | CATSPERG |
| Case 1 | 1 | 2 | utr3p | intron | KPNA6 | IRS1 |
| Case 1 | 21 | 12 | intron | upstream | AP001347.6 | AC079949.1 |
| Case 1 | 2 | 12 | intron | utr3p | SPP2 | C12orf50 |
| Case 1 | 5 | 2 | downstream | intron | CARTPT | PID1 |
| Case 1 | 8 | 8 | downstream | upstream | LY6E | C8orf31 |
| Case 1 | 11 | 11 | intron | upstream | DCPS | ST3GAL4 |
| Case 1 | 19 | 19 | intron | upstream | LINC00085 | MIR99B |
| Case 1 | 16 | 15 | utr3p | utr3p | RPS15A | BLOC1S6 |
| Case 1 | 19 | 19 | coding | upstream | KDELR1 | GRIN2D |
| Case 1 | 2 | 2 | downstream | intron | AC097532.2 | PID1 |
| Case 1 | 11 | 11 | intron | downstream | RP11-326C3.11 | RP11-326C3.10 |
| Case 1 | 1 | 8 | utr3p | utr3p | GABPB2 | PLEKHA2 |
| Case 1 | 1 | 3 | utr3p | utr3p | ZNF124 | SKIL |
| Case 1 | 16 | 8 | downstream | intron | RP11-14K3.2 | E2F5 |
| Case 1 | 1 | 6 | coding | intron | MEAF6 | PHF1 |
| Case 1 | 13 | 8 | intron | utr3p | LINC00355 | KIAA1456 |
| Case 1 | 11 | 11 | coding | intron | PEX16 | GYLTL1B |
| Case 1 | X | 1 | coding | intron | GNG5P2 | CTBS |
| Case 1 | 6 | 6 | intron | downstream | RP11-457M11.5 | GUSBP2 |
| Case 1 | 7 | 11 | utr3p | intron | ACTB | NXPE1 |
| Case 1 | 11 | 5 | utr3p | upstream | PCNXL3 | GRIA1 |
| Case 1 | 15 | 10 | utr3p | upstream | TJP1 | CCDC7 |
| Case 1 | 14 | X | coding | coding | NUBPL | PPP1R3F |
| Case 1 | 1 | 1 | utr3p | upstream | CCBL2 | RP11-82K18.2 |
| Case 1 | 19 | 19 | coding | upstream | KCNK6 | CATSPERG |
| Case 1 | 22 | 14 | utr3p | utr3p | XPNPEP3 | TMED10 |
| Case 1 | 20 | 20 | utr3p | utr3p | TTPAL | STX16 |
| Case 1 | 11 | 1 | utr3p | downstream | TAF1D | SNORA40 |
| Case 1 | 17 | 13 | utr3p | utr3p | LEPREL4 | FAM123A |
| Case 1 | 14 | 14 | utr3p | intron | PAPLN | NUMB |
| Case 1 | 16 | 4 | utr3p | intron | ZNF500 | RP11-58H15.1 |
| Case 1 | 3 | 4 | intron | downstream | STT3B | RP11-747H12.4 |
| Case 1 | 2 | 2 | coding | utr3p | LY75-CD302 | 07-Mar |
| Case 2 | 16 | 16 | coding | coding | RP11-680G10.1 | KIAA0182 |
| Case 2 | X | X | intron | coding | CD99P1 | CD99 |
| Case 2 | 16 | 16 | coding | coding | KIAA0182 | RP11-680G10.1 |
| Case 2 | 14 | 14 | utr5p | intron | RABGGTA | DHRS1 |
| Case 2 | 4 | 4 | utr5p | downstream | KIAA1211 | CEP135 |
| Case 2 | 16 | 16 | intron | coding | Z97634.5 | NME4 |
| Case 2 | 9 | 9 | coding | intron | XPA | NCBP1 |
| Case 2 | 9 | 9 | utr5p | upstream | SEC16A | C9orf163 |
| Case 2 | 1 | 1 | intron | intron | RP11-488L18.8 | RP11-488L18.4 |
| Case 2 | 1 | 1 | utr5p | downstream | SDF4 | FAM132A |
| Case 2 | 11 | 11 | coding | utr5p | SIDT2 | TAGLN |
| Case 2 | 16 | 16 | intron | utr5p | CTC-786C10.1 | RP11-680G10.1 |
| Case 2 | 3 | 3 | intron | upstream | RPL32P3 | RP11-529F4.1 |
| Case 2 | 11 | 11 | intron | intron | RP11-820L6.1 | RP11-166D19.1 |
| Case 2 | 19 | 19 | coding | upstream | ZNF544 | ZNF8 |
| Case 2 | 14 | 14 | utr5p | coding | RABGGTA | DHRS1 |
| Case 2 | 7 | 7 | coding | coding | TRBJ2-3 | TRBC2 |
| Case 2 | 7 | 7 | coding | coding | TRBJ2-3 | TRBC2 |
| Case 2 | 19 | 19 | coding | downstream | SLC1A5 | FKRP |
| Case 2 | X | 10 | utr5p | intron | FAM122C | RP11-90J7.3 |
| Case 2 | 22 | 22 | coding | downstream | TTLL12 | TTLL12 |
| Case 2 | 11 | 11 | coding | utr5p | POLA2 | CDC42EP2 |
| Case 2 | 7 | 17 | coding | coding | JAZF1 | SUZ12 |
| Case 2 | 1 | 1 | intron | upstream | SLC6A9 | RP5-1198O20.4 |
| Case 2 | 22 | 22 | intron | downstream | LL22NC03-80A10.6 | LL22NC03-2H8.4 |
| Case 2 | 4 | 2 | coding | coding | CFI | THADA |
| Case 2 | 9 | 9 | coding | coding | EDF1 | PHPT1 |
| Case 2 | 2 | 2 | coding | downstream | NFE2L2 | HNRNPA3 |
| Case 2 | 19 | 19 | coding | downstream | CFD | MED16 |
| Case 2 | 3 | 3 | intron | upstream | RP11-95M5.1 | FGD5 |
| Case 2 | 1 | 1 | coding | intron | MRPL37 | RP5-997D24.5 |
| Case 2 | 8 | 8 | upstream | intron | RP11-527N22.2 | RP11-150O12.1 |
| Case 2 | 2 | 1 | intron | intron | AC116050.1 | RP11-14N7.2 |
| Case 2 | 5 | 5 | intron | intron | RP11-141O11.2 | CTC-498J12.1 |
| Case 2 | 2 | 2 | coding | coding | ATP6V1B1 | VAX2 |
| Case 2 | 6 | 6 | coding | downstream | PRDM1 | 7SK |
| Case 2 | 20 | 20 | utr3p | utr3p | NCOA3 | SULF2 |
| Case 2 | 3 | 10 | intron | intron | RP11-413E6.1 | KIAA0664P5 |
| Case 2 | 6 | 6 | coding | downstream | PTCHD4 | HNRNPA3P4 |
| Case 2 | 17 | 17 | coding | downstream | AMZ2 | LOC440461 |
| Case 2 | 2 | 2 | utr5p | upstream | GREB1 | E2F6 |
| Case 2 | 2 | 1 | coding | intron | COL3A1 | RP5-857K21.4 |
| Case 2 | X | X | intron | intron | CHST7 | SLC9A7 |
| Case 2 | 4 | 4 | coding | upstream | CFI | PLA2G12A |
| Case 2 | 3 | 3 | utr5p | downstream | ALG1L | ROPN1B |
| Case 2 | 7 | 17 | coding | coding | JAZF1 | SUZ12 |
| Case 2 | 2 | 2 | intron | intron | AC068491.1 | AC108463.1 |
| Case 2 | 2 | 1 | coding | intron | COL3A1 | RP5-857K21.4 |
| Case 2 | 15 | 15 | coding | intron | NR2F2 | RP11-522B15.4 |
| Case 2 | 2 | 1 | intron | intron | AC116050.1 | RP11-14N7.2 |
| Case 2 | 20 | 20 | coding | intron | FRG1B | RP11-348I14.4 |
| Case 2 | 11 | 2 | coding | intron | LSP1 | AC116050.1 |
| Case 2 | 6 | 9 | intron | intron | MTO1 | EEF1A1P5 |
| Case 2 | 7 | 7 | coding | upstream | TRBJ2-5 | TRBC2 |
| Case 2 | 1 | 5 | coding | intron | ATAD3A | SKIV2L2 |
| Case 2 | 19 | 2 | downstream | intron | RNA5-8SP4 | PID1 |
| Case 2 | X | X | coding | upstream | HDHD1 | STS |
| Case 2 | 1 | 1 | coding | utr5p | SLC35E2 | CDK11A |
| Case 2 | 19 | 19 | utr3p | upstream | AC010642.1 | ZSCAN22 |
| Case 2 | 4 | 4 | utr5p | downstream | SH3D19 | U6 |
| Case 2 | 19 | 19 | utr3p | upstream | AC010642.1 | ZSCAN22 |
| Case 2 | 10 | 10 | intron | intron | RP11-773D16.1 | RP11-322M19.1 |
| Case 2 | 15 | 1 | intron | intron | GABRB3 | GAS5 |
| Case 2 | 10 | 10 | coding | intron | CRTAC1 | GOLGA7B |
| Case 2 | 1 | 1 | utr5p | upstream | PAQR7 | Y_RNA |
| Case 2 | 19 | 19 | utr5p | intron | GRIK5 | ZNF574 |
| Case 2 | 9 | 9 | coding | intron | HIATL1 | RP11-330M2.7 |
| Case 2 | 4 | 4 | coding | upstream | CFI | PLA2G12A |
| Case 2 | 6 | 1 | intron | intron | MDN1 | GAS5 |
| Case 2 | 2 | 2 | coding | intron | ACYP2 | AC008280.5 |
| Case 2 | 15 | 15 | coding | intron | RASL12 | SLC51B |
| Case 2 | 15 | 15 | intron | intron | RP13-608F4.8 | RP11-114H24.2 |
| Case 2 | 19 | 6 | utr3p | intron | FUT6 | RP11-704J17.5 |
| Case 2 | 6 | 5 | upstream | upstream | OR4F7P | AC138031.1 |
| Case 2 | 6 | 1 | intron | intron | MDN1 | GAS5 |
| Case 2 | 5 | 2 | downstream | intron | CARTPT | PID1 |
| Case 2 | 19 | 19 | coding | utr5p | CTB-133G6.1 | ARHGEF18 |
| Case 3 | 6 | 6 | utr5p | utr5p | PHF1 | ZBTB9 |
| Case 3 | 16 | 16 | utr5p | upstream | CHST6 | RP11-77K12.4 |
| Case 3 | 2 | 2 | coding | intron | ANO7 | HDLBP |
| Case 3 | 10 | 16 | utr5p | intron | SGMS1 | CHD9 |
| Case 3 | 21 | 21 | coding | intron | YBEY | C21orf58 |
| Case 3 | 2 | 18 | coding | upstream | PXDN | ATP9B |
| Case 3 | 7 | 7 | coding | coding | TRBJ2-4 | TRBC2 |
| Case 3 | 19 | 19 | intron | upstream | LINC00085 | MIR99B |
| Case 3 | X | X | coding | upstream | KDM5C | RP11-258C19.4 |
| Case 3 | 10 | 10 | coding | intron | CRTAC1 | GOLGA7B |
| Case 3 | 8 | 5 | utr5p | coding | HGSNAT | BHMT |
| Case 3 | 1 | 1 | coding | upstream | LMOD1 | RP11-307B6.3 |
| Case 3 | 19 | 2 | downstream | intron | RNA5-8SP4 | PID1 |
| Case 3 | 7 | 7 | upstream | intron | TWIST1 | AC003986.7 |
| Case 3 | 9 | 9 | coding | intron | XPA | NCBP1 |
| Case 3 | 7 | 7 | intron | intron | PMS2P3 | TYW1 |
| Case 3 | 12 | 17 | utr5p | intron | AMDHD1 | BRCA1 |
| Case 3 | 2 | 2 | intron | intron | AC116050.1 | AC027612.6 |
| Case 3 | 14 | 14 | utr3p | intron | PAPLN | NUMB |
| Case 3 | 1 | 1 | downstream | intron | RP5-924G13.2 | GAS5 |
| Case 3 | 12 | 12 | intron | intron | THAP2 | RP11-293I14.2 |
| Case 3 | 5 | 5 | intron | downstream | FAM153C | RP11-889L3.1 |
| Case 3 | 11 | 2 | coding | intron | LSP1 | AC116050.1 |
| Case 3 | 17 | 6 | intron | intron | ZNF652 | SNHG5 |
| Case 3 | 19 | 19 | utr3p | upstream | AC010642.1 | ZSCAN22 |
| Case 3 | 6 | 1 | intron | intron | MDN1 | GAS5 |
| Case 3 | 6 | 1 | intron | intron | MDN1 | GAS5 |
| Case 3 | 19 | 19 | upstream | utr3p | CD320 | NDUFA7 |
| Case 3 | 2 | 2 | coding | utr5p | KLHL23 | SSB |
| Case 3 | 9 | 9 | intron | upstream | RP11-262H14.3 | RP11-104G3.2 |
| Case 3 | 9 | 2 | upstream | intron | AL161626.1 | PKP4 |
| Case 3 | 17 | 6 | intron | intron | ZNF652 | SNHG5 |
| Case 3 | 2 | 2 | coding | intron | ACYP2 | AC008280.5 |
| Case 3 | 11 | 2 | coding | intron | LSP1 | AC116050.1 |
| Case 3 | 14 | 14 | upstream | intron | WDR89 | U3 |
| Case 3 | 14 | 14 | utr5p | intron | RABGGTA | DHRS1 |
| Case 3 | 17 | 4 | utr5p | intron | BCAS3 | RP11-44F21.5 |
| Case 3 | 6 | 4 | utr3p | intron | SYNGAP1 | RP11-425A23.1 |
| Case 3 | 2 | 2 | utr3p | intron | SUPT7L | GPN1 |
| Case 3 | 6 | 6 | intron | upstream | RP11-457M11.5 | XXbac-BPG55C20.6 |
| Case 3 | 14 | 14 | intron | downstream | RP11-529H20.6 | RP11-529H20.5 |
| Case 3 | 22 | 17 | upstream | intron | CRKL | UNC45B |
| Case 3 | 5 | 5 | intron | intron | RP11-141O11.2 | CTC-498J12.1 |
| Case 3 | 4 | 7 | coding | coding | TMEM184C | TAX1BP1 |
| Case 3 | 8 | 16 | intron | downstream | E2F5 | RP11-14K3.2 |
| Case 3 | 19 | 19 | coding | intron | ZNF814 | ZNF587 |
| Case 3 | 3 | 3 | intron | intron | RP11-271E2.1 | RP11-95M5.1 |
| Case 3 | 22 | 7 | utr3p | utr3p | FAM227A | DGKB |
| Case 3 | 4 | 1 | intron | intron | PPEF2 | HFM1 |
| Case 3 | 19 | 19 | coding | upstream | ZNF544 | ZNF8 |
| Case 3 | 19 | 19 | downstream | coding | PNMAL2 | PPP5D1 |
| Case 3 | 6 | 1 | intron | intron | MDN1 | GAS5 |
| Case 3 | 19 | 19 | utr3p | upstream | AC010642.1 | ZSCAN22 |
| Case 3 | 1 | 1 | coding | intron | RWDD3 | RP11-57H12.5 |
| Case 3 | 2 | 2 | upstream | coding | Y_RNA | AC007405.7 |
| Case 3 | 3 | 3 | downstream | intron | AC092902.1 | RP11-379B18.4 |
| Case 3 | 15 | 1 | intron | intron | GABRB3 | GAS5 |
| Case 3 | 2 | 2 | downstream | intron | AC097532.2 | PID1 |
| Case 3 | 9 | 9 | coding | downstream | HIATL1 | FAM22F |
| Case 3 | 12 | 2 | downstream | coding | RP11-467L13.4 | DPY30 |
| Case 3 | 4 | 15 | utr3p | intron | FAM114A1 | RP11-483E23.2 |
| Case 3 | 4 | X | coding | utr5p | FRG1 | FAM122C |
| Case 3 | 19 | 11 | intron | intron | FUT2 | RNH1 |
| Case 3 | 19 | 10 | intron | utr3p | ZNF761 | VSTM4 |
| Case 3 | 10 | 22 | coding | downstream | BMS1 | LL22NC03-2H8.4 |
| Case 3 | 19 | 19 | utr3p | downstream | EMC10 | JOSD2 |
| Case 3 | 2 | 1 | intron | intron | AC116050.1 | RP11-14N7.2 |
| Case 3 | 17 | 19 | utr5p | intron | SLC52A1 | AKT2 |
| Case 3 | 19 | 3 | intron | utr3p | CEACAM22P | GTF2E1 |
| Case 3 | 1 | 19 | utr3p | utr3p | GPR157 | ZNF264 |
| Case 3 | 10 | X | intron | utr3p | RP11-508M1.3 | CXorf23 |
| Case 3 | 3 | 22 | utr3p | utr3p | ZNF620 | DESI1 |
| Case 3 | 17 | 6 | intron | intron | ZNF652 | SNHG5 |
| Case 3 | 4 | 3 | intron | intron | SCD5 | AC069513.3 |
| Case 3 | 22 | 3 | utr3p | utr3p | DMC1 | KBTBD12 |
| Case 3 | 18 | 18 | intron | coding | RP11-751H17.1 | GALR1 |
| Case 3 | 5 | 8 | intron | upstream | SSBP2 | U2 |
| Case 3 | 10 | 18 | coding | intron | MGEA5 | TYMS |
| Case 3 | 3 | 22 | intron | intron | RP11-280F2.2 | TPTEP1 |
| Case 3 | 1 | 15 | utr3p | utr5p | CYB561D1 | C15orf63 |
| Case 3 | 21 | 20 | utr5p | utr5p | TTC3 | GDF5 |
| Case 3 | 6 | 1 | intron | intron | MDN1 | GAS5 |
| Case 3 | 5 | 6 | utr3p | intron | DNAJC21 | BTBD9 |
| Case 3 | 7 | 6 | coding | utr5p | JAZF1 | PHF1 |
| Case 4 | 11 | 11 | coding | intron | EED | RP11-320L11.2 |
| Case 4 | 2 | 2 | intron | upstream | AC007386.4 | CEP68 |
| Case 4 | 5 | 12 | intron | intron | CTD-2066L21.3 | RP11-705C15.2 |
| Case 4 | 1 | 1 | utr3p | downstream | AIM2 | AL359753.1 |
| Case 4 | 2 | 2 | coding | downstream | TGFBRAP1 | GPR45 |
| Case 4 | 19 | 19 | coding | upstream | ATP13A1 | ZNF101 |
| Case 4 | 2 | 2 | coding | coding | PPP1R21 | AC073082.1 |
| Case 4 | 1 | 9 | intron | utr5p | SNHG3 | PDCD1LG2 |
| Case 4 | 6 | 6 | coding | intron | CNPY3 | RP3-475N16.1 |
| Case 4 | 11 | 11 | coding | downstream | GRAMD1B | AP000783.2 |
| Case 4 | 5 | 5 | intron | downstream | HINT1 | LYRM7 |
| Case 4 | 7 | 7 | upstream | coding | TRBJ2-1 | TRBC2 |
| Case 4 | 1 | 9 | intron | coding | SNHG3 | PDCD1LG2 |
| Case 4 | 4 | 4 | intron | upstream | HERC6 | HERC5 |
| Case 4 | 14 | 14 | utr5p | intron | RABGGTA | DHRS1 |
| Case 4 | 1 | 1 | utr5p | coding | TNFAIP8L2 | SCNM1 |
| Case 4 | 5 | 5 | intron | downstream | HINT1 | LYRM7 |
| Case 4 | 1 | 1 | coding | downstream | PMVK | PBXIP1 |
| Case 4 | 6 | 2 | intron | intron | RP5-1120P11.1 | AC013463.2 |
| Case 4 | 12 | 12 | coding | upstream | CLECL1 | RP11-75L1.1 |
| Case 4 | 9 | 9 | intron | downstream | COL5A1 | RP11-263F14.3 |
| Case 4 | 19 | 19 | coding | intron | ZNF814 | ZNF587 |
| Case 4 | 22 | 1 | utr5p | coding | GGT1 | IGSF3 |
| Case 4 | 1 | 1 | utr5p | upstream | PRDX1 | AKR1A1 |
| Case 4 | 3 | 12 | coding | intron | AC103588.1 | RP11-705C15.2 |
| Case 4 | 11 | 11 | intron | upstream | RP11-286N22.10 | MIR4488 |
| Case 4 | 9 | 9 | utr5p | upstream | SEC16A | C9orf163 |
| Case 4 | 2 | 2 | upstream | coding | Y_RNA | AC007405.7 |
| Case 4 | 14 | 14 | coding | coding | TRAJ12 | TRAC |
| Case 4 | 14 | 14 | coding | coding | TRAJ9 | TRAC |
| Case 4 | 7 | 7 | coding | coding | TRBJ2-3 | TRBC2 |
| Case 4 | 12 | 12 | coding | downstream | YEATS4 | Metazoa_SRP |
| Case 4 | 12 | 12 | coding | upstream | SBNO1 | RP13-942N8.1 |
| Case 4 | 2 | 2 | intron | upstream | AC007386.4 | CEP68 |
| Case 4 | 7 | 7 | intron | intron | AC004967.8 | AC004967.7 |
| Case 4 | 11 | 11 | coding | intron | CTSC | RAB38 |
| Case 4 | 2 | 2 | upstream | intron | WDFY1 | MRPL44 |
| Case 4 | 9 | 9 | intron | downstream | COL5A1 | RP11-263F14.3 |
| Case 4 | 2 | 2 | upstream | intron | WDFY1 | MRPL44 |
| Case 4 | 2 | 2 | coding | intron | IGKV5-2 | IGKJ5 |
| Case 4 | 11 | 11 | intron | intron | NLRP6 | RP11-326C3.2 |
| Case 4 | 17 | 17 | upstream | intron | AC104024.1 | AC104024.2 |
| Case 4 | 1 | 1 | downstream | intron | ELL2P1 | CD1D |
| Case 4 | 13 | 6 | coding | downstream | RB1 | GPR6 |
| Case 4 | 7 | 7 | coding | coding | TRBJ2-4 | TRBC2 |
| Case 4 | 19 | 19 | coding | upstream | ZNF544 | ZNF8 |
| Case 4 | 7 | 3 | intron | intron | AC005682.5 | MAP4 |
| Case 4 | 14 | 14 | coding | downstream | GZMB | RP11-104E19.1 |
| Case 4 | 14 | 14 | downstream | upstream | TRAV13-1 | TRAC |
| Case 4 | 12 | 2 | intron | utr3p | CDK17 | NCL |
| Case 4 | 19 | 19 | utr3p | upstream | AC010642.1 | ZSCAN22 |
| Case 4 | 6 | 1 | intron | intron | MDN1 | GAS5 |
| Case 4 | 15 | 1 | intron | intron | GABRB3 | GAS5 |
| Case 4 | 3 | 3 | utr3p | coding | ACTR8 | IL17RB |
| Case 4 | 10 | 10 | intron | utr3p | RP11-310E22.5 | C10orf129 |
| Case 4 | 17 | 17 | coding | coding | NSF | LRRC37A3 |
| Case 4 | 12 | 12 | intron | downstream | RP11-554D14.4 | RP11-554D14.2 |
| Case 4 | 20 | 20 | coding | intron | SIRPG | RP4-673D20.1 |
| Case 4 | 10 | 10 | intron | intron | RP11-773D16.1 | RP11-322M19.1 |
| Case 4 | 1 | 1 | intron | downstream | RP11-488L18.4 | RP11-488L18.8 |
| Case 4 | 6 | 1 | intron | intron | MDN1 | GAS5 |
| Case 4 | 17 | 6 | intron | intron | ZNF652 | SNHG5 |
| Case 4 | 12 | 12 | coding | upstream | CLECL1 | RP11-75L1.1 |
| Case 4 | 12 | 12 | coding | downstream | KLRB1 | U6 |
| Case 4 | 19 | 19 | utr3p | upstream | AC010642.1 | ZSCAN22 |
| Case 4 | 6 | 1 | intron | intron | MDN1 | GAS5 |
| Case 4 | 2 | 2 | coding | utr5p | RRM2 | C2orf48 |
| Case 4 | 15 | 15 | utr3p | utr3p | SPG21 | ANKDD1A |
| Case 4 | 18 | 12 | intron | intron | KIAA1328 | CD163 |
| Case 4 | 7 | 7 | coding | coding | TRBJ2-2 | TRBC2 |
| Case 4 | 17 | 13 | intron | intron | MYH13 | ATP8A2 |
| Case 4 | 5 | 5 | utr3p | downstream | ATOX1 | CTB-113P19.1 |
| Case 4 | 5 | 5 | coding | downstream | GZMA | GZMAP1 |
| Case 4 | 14 | 14 | coding | intron | IGHG2 | RP11-731F5.2 |
| Case 4 | 1 | 1 | utr5p | downstream | UTS2 | TNFRSF9 |
| Case 4 | 17 | 6 | intron | intron | ZNF652 | SNHG5 |
| Case 4 | 8 | 5 | intron | coding | AF121898.3 | NAIP |
| Case 4 | 14 | 14 | utr5p | intron | KIAA0125 | AL122127.25 |
| Case 4 | 11 | 11 | downstream | intron | CD6 | RP11-881M11.8 |
| Case 4 | 7 | 5 | utr3p | downstream | TRA2A | PRR16 |
| Case 4 | 10 | 10 | intron | intron | RP11-773D16.1 | RP11-119F19.2 |
| Case 4 | 16 | 16 | downstream | intron | CTD-2576D5.3 | XYLT1 |
| Case 4 | 11 | 11 | intron | upstream | DCPS | ST3GAL4 |
| Case 4 | 17 | 17 | coding | upstream | RPL23 | LASP1 |
| Case 4 | 5 | 5 | coding | intron | NAIP | RP11-974F13.6 |
| Case 4 | 5 | 2 | intron | downstream | RP11-267A15.1 | U6 |
| Case 4 | 15 | 1 | intron | intron | GABRB3 | GAS5 |
| Case 4 | 7 | 3 | intron | intron | AC005682.6 | MAP4 |
| Case 4 | 17 | 17 | intron | intron | PPP1R9B | RP11-893F2.13 |
| Case 4 | 12 | 2 | intron | intron | RP11-513G19.1 | SCN9A |
| Case 4 | 6 | 6 | coding | downstream | FABP7 | Metazoa_SRP |
| Case 4 | 17 | 18 | utr5p | downstream | TMEM98 | CDH2 |
| Case 4 | 15 | 1 | intron | intron | GABRB3 | GAS5 |
| Case 4 | 22 | 22 | intron | downstream | LL22NC03-80A10.6 | LL22NC03-2H8.4 |
| Case 4 | 14 | 14 | coding | upstream | IGHG4 | IGHG1 |
| Case 4 | 7 | 7 | coding | coding | TRBJ2-7 | TRBC2 |
| Case 5 | 1 | 1 | coding | coding | PLEKHO1 | VPS45 |
| Case 5 | X | X | utr5p | utr3p | ARMCX1 | ARMCX4 |
| Case 5 | 9 | 9 | coding | intron | XPA | NCBP1 |
| Case 5 | 8 | 8 | utr5p | intron | DSCC1 | KB-1471A8.1 |
| Case 5 | 14 | 14 | downstream | coding | TRAJ23 | TRAC |
| Case 5 | 7 | 7 | coding | intron | TMEM196 | AC004543.2 |
| Case 5 | 7 | 7 | coding | upstream | ITGB8 | EEF1A1P27 |
| Case 5 | 19 | 19 | intron | upstream | LINC00085 | MIR99B |
| Case 5 | 19 | 19 | utr3p | upstream | NDUFA7 | CD320 |
| Case 5 | 14 | 14 | utr5p | intron | RABGGTA | DHRS1 |
| Case 5 | 19 | 19 | coding | upstream | KCNK6 | CATSPERG |
| Case 5 | 14 | 14 | coding | intron | KLHDC2 | NEMF |
| Case 5 | 7 | 7 | downstream | intron | AC091320.2 | RP11-328J2.1 |
| Case 5 | 7 | 7 | downstream | intron | AC091320.2 | RP11-328J2.1 |
| Case 5 | 19 | 19 | coding | upstream | ZNF544 | ZNF8 |
| Case 5 | 2 | 1 | intron | intron | AC116050.1 | RP11-14N7.2 |
| Case 5 | 17 | 17 | intron | intron | BZRAP1 | BZRAP1-AS1 |
| Case 5 | 10 | 10 | coding | intron | SEC31B | WNT8B |
| Case 5 | 7 | 7 | upstream | intron | AC091320.2 | RP11-328J2.1 |
| Case 5 | 14 | 14 | downstream | coding | TRAJ23 | TRAC |
| Case 5 | 14 | 14 | downstream | coding | TRAJ23 | TRAC |
| Case 5 | 1 | 1 | utr5p | upstream | S100A7 | Metazoa_SRP |
| Case 5 | 10 | 10 | coding | intron | DLG5 | RP11-126H7.3 |
| Case 5 | X | 15 | utr3p | downstream | TBC1D8B | CTD-2054N24.2 |
| Case 5 | 11 | 11 | downstream | intron | RP11-405K6.1 | RP11-20J1.1 |
| Case 5 | 12 | 12 | intron | intron | RFC5 | WSB2 |
| Case 5 | 5 | 1 | coding | upstream | AC138035.2 | RP11-54O7.1 |
| Case 5 | 22 | 22 | coding | coding | ARVCF | COMT |
| Case 5 | 17 | 7 | intron | downstream | RP11-334C17.5 | AKR1B1 |
| Case 5 | 9 | 9 | utr3p | intron | SLC25A25 | RP11-395P17.3 |
| Case 5 | 22 | 22 | utr3p | coding | MICALL1 | POLR2F |
| Case 5 | 1 | 1 | coding | downstream | AMY1B | AMY2B |
| Case 5 | 2 | 2 | utr3p | intron | CFLAR | AC007283.5 |
| Case 5 | 9 | 9 | coding | coding | SUSD1 | PTBP3 |
| Case 5 | 17 | 6 | intron | intron | ZNF652 | SNHG5 |
| Case 5 | 2 | 2 | coding | upstream | AC007405.7 | Y_RNA |
| Case 5 | 3 | 6 | coding | upstream | RPL24 | EEF1E1 |
| Case 5 | 5 | 5 | coding | downstream | DBN1 | PDLIM7 |
| Case 5 | 1 | 1 | utr3p | intron | TARDBP | MASP2 |
| Case 5 | 16 | 14 | utr3p | utr5p | MEFV | BRF1 |
| Case 5 | 1 | 1 | coding | utr5p | SLC35E2 | CDK11A |
| Case 5 | 7 | 7 | upstream | intron | AC091320.2 | RP11-328J2.1 |
| Case 5 | 19 | 2 | downstream | intron | RNA5-8SP4 | PID1 |
| Case 5 | 19 | 19 | coding | upstream | ZNF544 | ZNF8 |
| Case 5 | 15 | 15 | utr3p | utr3p | SPG21 | ANKDD1A |
| Case 5 | 4 | 4 | intron | intron | RP11-168E14.1 | BMPR1B |
| Case 5 | 6 | 6 | utr3p | intron | PLA2G7 | RP11-446F17.3 |
| Case 5 | 1 | 11 | intron | upstream | NFASC | RELA |
| Case 5 | 4 | 4 | upstream | intron | RP11-728C8.1 | CTD-2325B11.1 |
| Case 5 | 2 | 2 | coding | utr5p | KLHL23 | SSB |
| Case 5 | 6 | X | intron | downstream | FAM217A | RP11-232D9.1 |
| Case 5 | 22 | 22 | intron | downstream | LL22NC03-80A10.6 | LL22NC03-2H8.4 |
| Case 5 | 2 | 2 | coding | intron | ANAPC1 | AC125232.1 |
| Case 5 | 6 | 1 | intron | intron | MDN1 | GAS5 |
| Case 5 | 2 | 2 | intron | upstream | AC018717.1 | AC018717.2 |
| Case 5 | 16 | 16 | intron | coding | Z97634.5 | NME4 |
| Case 5 | 19 | 19 | utr3p | upstream | AC010642.1 | ZSCAN22 |
| Case 5 | 10 | 10 | utr3p | upstream | TIMM23B | AGAP6 |
| Case 5 | 17 | 6 | intron | intron | ZNF652 | SNHG5 |
| Case 5 | 5 | 5 | intron | intron | SDHAP3 | RP11-43F13.1 |
| Case 5 | 12 | 12 | coding | downstream | KLRB1 | U6 |
| Case 5 | 11 | 11 | coding | utr5p | PCSK7 | TAGLN |
| Case 5 | 1 | 8 | utr5p | intron | DISP1 | VPS13B |
| Case 5 | 7 | 7 | upstream | intron | EEF1A1P27 | AC002486.3 |
| Case 5 | X | X | downstream | downstream | TAZ | BX936347.1 |
| Case 5 | 2 | 2 | upstream | upstream | HIGD1AP4 | AC013448.1 |
| Case 5 | 2 | 2 | intron | upstream | AC018717.1 | U7 |
| Case 5 | 11 | 2 | coding | intron | LSP1 | AC116050.1 |
| Case 5 | 8 | 4 | intron | upstream | RP11-556O5.3 | RP11-180A12.1 |
| Case 5 | 5 | 5 | intron | upstream | SDHAP3 | CTD-2012J19.2 |
| Case 5 | 4 | 4 | upstream | intron | SNORA70 | RP11-614F17.2 |
| Case 5 | 4 | 1 | intron | intron | PPEF2 | HFM1 |
| Case 5 | 17 | 17 | utr5p | downstream | SPACA3 | TMEM98 |
| Case 5 | 5 | 5 | intron | intron | RP11-43F13.1 | SDHAP3 |
| Case 5 | 6 | 1 | intron | intron | MDN1 | GAS5 |
| Case 5 | 20 | 7 | intron | upstream | LINC00652 | AC091320.2 |
| Case 5 | 6 | 1 | intron | intron | MDN1 | GAS5 |
| Case 5 | 7 | 7 | intron | coding | AC017116.8 | STAG3L3 |
| Case 5 | 19 | 19 | utr3p | upstream | AC010642.1 | ZSCAN22 |
| Case 5 | 10 | 10 | intron | upstream | RP11-773D16.1 | RP11-399L7.2 |
| Case 5 | 1 | 1 | utr3p | utr5p | SMG5 | PAQR6 |
| Case 5 | 7 | 9 | intron | intron | AC074183.4 | SNHG7 |
| Case 5 | 2 | 2 | downstream | intron | IGKV3-20 | IGKJ5 |
| Case 5 | 1 | 1 | downstream | upstream | RP3-395M20.7 | RP3-395M20.9 |
| Case 5 | 3 | 3 | intron | downstream | MYRIP | ENTPD3-AS1 |
| Case 5 | 2 | 2 | utr3p | intron | CFLAR | AC007283.5 |
| Case 5 | 22 | 22 | coding | upstream | C22orf43 | IGLL1 |
| Case 5 | 22 | 22 | coding | coding | IGLL5 | IGLV1-40 |
| Case 5 | 6 | 1 | intron | intron | MDN1 | GAS5 |
| Case 5 | 16 | 16 | intron | intron | HS3ST4 | SRCAP |
| Case 5 | 2 | 2 | coding | utr5p | RRM2 | C2orf48 |
| Case 5 | 22 | 5 | intron | coding | RP1-130H16.16 | SQSTM1 |
| Case 5 | 7 | 7 | upstream | utr5p | EEF1A1P27 | ABCB5 |
| Case 5 | 1 | 1 | utr3p | intron | TMEM63A | EPHX1 |
| Case 5 | 16 | 16 | utr3p | intron | EIF3C | PDXDC2P |
| Case 5 | 6 | 1 | intron | intron | MDN1 | GAS5 |
| Case 5 | 1 | 1 | utr3p | upstream | CCBL2 | RP11-82K18.2 |
| Case 5 | 15 | 1 | intron | intron | GABRB3 | GAS5 |
| Case 5 | 12 | 12 | utr3p | downstream | ESYT1 | RP11-603J24.14 |
| Case 5 | 16 | 16 | utr3p | intron | CIITA | DEXI |
| Case 5 | 5 | 1 | coding | intron | AC138035.2 | RP11-54O7.1 |
| Case 5 | 6 | 10 | utr3p | utr3p | MMS22L | PDZD8 |
| Case 5 | 15 | 1 | intron | intron | GABRB3 | GAS5 |
| Case 5 | 8 | 7 | utr5p | downstream | RP11-481A20.11 | AC079781.8 |
| Case 5 | 3 | 16 | utr3p | intron | TMEM212 | ATF7IP2 |
| Case 5 | 5 | 1 | coding | intron | AC138035.2 | RP11-54O7.1 |
| Case 5 | 13 | 13 | intron | upstream | DLEU1 | GTF2F2 |
| Case 5 | 14 | 7 | coding | intron | RNASE10 | ABCB5 |
| Case 5 | 3 | 16 | utr3p | intron | LPP | BRD7 |
| Case 5 | 10 | 19 | utr3p | intron | REEP3 | TPM4 |
| Case 5 | X | X | utr5p | upstream | FAM9B | RP11-430A19.2 |
| Case 5 | 1 | 1 | downstream | intron | RP5-924G13.2 | GAS5 |
| Case 5 | 9 | 9 | downstream | intron | RP5-1050E16.1 | RP5-1050E16.2 |
| Case 5 | 1 | 13 | utr3p | intron | GSTM3 | CARS2 |
| Case 5 | 1 | 7 | intron | utr3p | RP11-261C10.3 | NUPR1L |
| Case 5 | 12 | 9 | coding | intron | AAAS | LINC00476 |
| Case 5 | 13 | 7 | intron | downstream | GPC5 | AKR1B1 |
| Case 5 | 9 | 9 | coding | intron | GOLM1 | NAA35 |
| Case 5 | 17 | 1 | utr3p | utr3p | VPS53 | IL28RA |
| Case 5 | 9 | 2 | upstream | intron | AL161626.1 | PKP4 |
| Case 5 | 2 | 2 | coding | intron | IGKV3-11 | IGKJ5 |
| Case 5 | 2 | 2 | utr3p | intron | SUPT7L | GPN1 |
| Case 5 | 14 | X | utr3p | utr3p | GNPNAT1 | PGK1 |
| Case 5 | 7 | 7 | coding | coding | ATP6V0E2 | ACTR3B |
| Case 5 | 16 | 1 | coding | intron | HYDIN | RP4-565E6.1 |
| Case 5 | 2 | 2 | coding | intron | ACYP2 | AC008280.5 |
| Case 5 | 2 | 1 | intron | utr3p | SOWAHC | TARS2 |
| Case 5 | 8 | 19 | utr3p | utr3p | MTMR9 | KIR3DX1 |
| Case 5 | 1 | 1 | downstream | intron | RP5-924G13.2 | GAS5 |
