# Supplementary material for: Genomic landscape of endometrial stromal sarcoma of uterus
Source: Oncotarget. 2015 Sep 30;6(32):33319–28. doi: 10.18632/oncotarget.5384 (PMC4741768; doi:10.18632/oncotarget.5384)
Supplement: Supplementary file 3 [file oncotarget-06-33319-s003.docx]

**Table S3. Somatic point mutations and indels identified across five endometrial stromal sarcoma genomes**

| **ID** | **Chr** | **Start** | **End** | **Gene** | **Reference sequence** | **Altered sequence** | **Amino acid change** | **Exonic function** | **Mutant allele frequency** |
| --- | --- | --- | --- | --- | --- | --- | --- | --- | --- |
| Case 1 | 1 | 21806710 | 21806710 | NBPF3 | T | G | NM_032264:p.L459V | nonsynonymous SNV | 0.16 |
| Case 1 | 1 | 21806667 | 21806667 | NBPF3 | C | G | NM_032264:p.D444E | nonsynonymous SNV | 0.08 |
| Case 1 | 2 | 231363252 | 231363252 | SP100 | T | TA | NM_001080391:c.1720+2T>TA | splicing | 0.30 |
| Case 1 | 10 | 101124187 | 101124187 | CNNM1 | T | G | NM_020348:p.V681G | nonsynonymous SNV | 0.17 |
| Case 1 | 10 | 101124184 | 101124184 | CNNM1 | A | G | NM_020348:p.E680G | nonsynonymous SNV | 0.16 |
| Case 1 | 12 | 132426131 | 132426131 | PUS1 | C | T | NM_025215:p.A280V | nonsynonymous SNV | 0.05 |
| Case 1 | 19 | 44778795 | 44778796 | ZNF233 | CG | C | NM_181756:c.1982_1983C | frameshift substitution | 0.93 |
| Case 2 | 6 | 26451927 | 26451927 | BTN3A3 | C | T | NM_006994:p.T348M | nonsynonymous SNV | 0.04 |
| Case 2 | 7 | 11441562 | 11441562 | THSD7A | C | T | NM_015204:p.S1424N | nonsynonymous SNV | 0.79 |
| Case 2 | 11 | 5274541 | 5274541 | HBG2 | C | G | NM_000184:p.G137A | nonsynonymous SNV | 0.10 |
| Case 2 | 16 | 48145574 | 48145574 | ABCC12 | C | T | NM_033226:c.2125-1G>A | splicing | 0.03 |
| Case 2 | 21 | 47862475 | 47862475 | PCNT | C | A | NM_006031:p.A3230D | nonsynonymous SNV | 0.05 |
| Case 2 | X | 100265639 | 100265639 | TRMT2B | C | G | NM_024917:p.Q482H | nonsynonymous SNV | 0.42 |
| Case 3 | 1 | 52732372 | 52732372 | ZFYVE9 | A | AC | NM_004799:c.2324_2324delinsAC | frameshift substitution | 0.47 |
| Case 3 | 3 | 161221409 | 161221410 | OTOL1 | GA | G | NM_001080440:c.1113_1114G | frameshift substitution | 0.48 |
| Case 3 | 3 | 9166506 | 9166506 | SRGAP3 | G | A | NM_014850:p.R55W | nonsynonymous SNV | 0.08 |
| Case 3 | 3 | 30898586 | 30898586 | GADL1 | C | A | NM_207359:p.L86F | nonsynonymous SNV | 0.44 |
| Case 3 | 3 | 126180638 | 126180638 | ZXDC | C | T | NM_025112:p.D623N | nonsynonymous SNV | 0.52 |
| Case 3 | 4 | 7012383 | 7012383 | TBC1D14 | C | A | NM_020773:p.Q508K | nonsynonymous SNV | 0.04 |
| Case 3 | 6 | 42797772 | 42797773 | GLTSCR1L | AG | A | NM_015349:c.1701_1702A | frameshift substitution | 0.43 |
| Case 3 | 6 | 80745132 | 80745132 | TTK | A | G | NM_003318:p.H641R | nonsynonymous SNV | 0.44 |
| Case 3 | 6 | 147057703 | 147057703 | ADGB | A | T | NM_024694:p.E955V | nonsynonymous SNV | 0.40 |
| Case 3 | 6 | 147057702 | 147057702 | ADGB | G | T | NM_024694:p.E955X | stopgain SNV | 0.40 |
| Case 3 | 10 | 76784945 | 76784949 | KAT6B | GACAA | G | NM_012330:c.3602_3606G | frameshift substitution | 0.41 |
| Case 3 | 10 | 3180251 | 3180251 | PITRM1 | G | T | NM_014889:p.A1029D | nonsynonymous SNV | 0.04 |
| Case 3 | 10 | 7679296 | 7679296 | ITIH5 | C | T | NM_030569:p.G183R | nonsynonymous SNV | 0.40 |
| Case 3 | 11 | 205345 | 205345 | BET1L | G | A | NM_001098787:p.A98V | nonsynonymous SNV | 0.49 |
| Case 3 | 12 | 46594889 | 46594889 | SLC38A1 | G | C | NM_001278387:p.T332R | nonsynonymous SNV | 0.56 |
| Case 3 | 19 | 56228238 | 56228239 | NLRP9 | TC | T | NM_176820:c.2185_2186A | frameshift substitution | 0.48 |
| Case 3 | 19 | 1466115 | 1466115 | APC2 | C | T | NM_005883:p.R939C | nonsynonymous SNV | 0.45 |
| Case 3 | 20 | 55027175 | 55027177 | CASS4 | CTT | C | NM_001164116:c.943_945C | frameshift substitution | 0.47 |
| Case 3 | 20 | 2616592 | 2616592 | TMC2 | A | T | NM_080751:p.N776I | nonsynonymous SNV | 0.54 |
| Case 3 | 20 | 2641610 | 2641610 | IDH3B | T | A | NM_174855:p.I115F | nonsynonymous SNV | 0.48 |
| Case 3 | 20 | 2739597 | 2739597 | EBF4 | G | T | NM_001110514:p.R590L | nonsynonymous SNV | 0.52 |
| Case 3 | X | 53966729 | 53966729 | PHF8 | C | T | NM_015107:p.R957H | nonsynonymous SNV | 0.45 |
| Case 4 | 2 | 151328250 | 151328250 | RND3 | C | A | NM_005168:p.C125F | nonsynonymous SNV | 0.19 |
| Case 4 | 3 | 38051877 | 38051877 | PLCD1 | G | A | NM_006225:p.P323S | nonsynonymous SNV | 0.04 |
| Case 4 | 3 | 32022484 | 32022484 | OSBPL10 | G | A | NM_017784:p.A63V | nonsynonymous SNV | 0.15 |
| Case 4 | 4 | 90035564 | 90035564 | TIGD2 | C | T | NM_145715:p.A480V | nonsynonymous SNV | 0.04 |
| Case 4 | 5 | 158526459 | 158526459 | EBF1 | G | A | NM_182708:p.R10W | nonsynonymous SNV | 0.09 |
| Case 4 | 6 | 393217 | 393217 | IRF4 | G | A | NM_002460:p.G22E | nonsynonymous SNV | 0.07 |
| Case 4 | 8 | 38067780 | 38067780 | BAG4 | A | C | NM_004874:p.K381N | nonsynonymous SNV | 0.09 |
| Case 4 | 13 | 33095573 | 33095573 | N4BP2L2 | C | T | NM_033111:p.G67E | nonsynonymous SNV | 0.09 |
| Case 4 | 22 | 23235960 | 23235960 | IGLL5 | G | C | NM_001178126:p.C96S | nonsynonymous SNV | 0.13 |
| Case 4 | X | 14862030 | 14862030 | FANCB | G | T | NM_001018113:p.L747I | nonsynonymous SNV | 0.08 |
| Case 5 | 1 | 154108392 | 154108392 | NUP210L | G | C | NM_207308:p.L303V | nonsynonymous SNV | 0.15 |
| Case 5 | 1 | 248814013 | 248814013 | OR2T27 | G | T | NM_001001824:p.P58H | nonsynonymous SNV | 0.21 |
| Case 5 | 2 | 43451630 | 43451630 | ZFP36L2 | G | A | NM_006887:p.S438F | nonsynonymous SNV | 0.47 |
| Case 5 | 2 | 80136918 | 80136918 | CTNNA2 | A | C | NM_004389:p.N351H | nonsynonymous SNV | 0.15 |
| Case 5 | 3 | 12434198 | 12434198 | PPARG | A | C | NM_138712:p.K161T | nonsynonymous SNV | 0.09 |
| Case 5 | 3 | 108163661 | 108163661 | MYH15 | C | A | NM_014981:p.K847N | nonsynonymous SNV | 0.37 |
| Case 5 | 4 | 88959446 | 88959447 | PKD2 | AG | A | NM_000297:c.887_888A | frameshift substitution | 0.42 |
| Case 5 | 4 | 3318887 | 3318887 | RGS12 | G | T | NM_198229:p.E330D | nonsynonymous SNV | 0.11 |
| Case 5 | 4 | 79205585 | 79205585 | FRAS1 | T | A | NM_025074:p.C428S | nonsynonymous SNV | 0.03 |
| Case 5 | 5 | 146658940 | 146658941 | STK32A | AC | A | NM_145001:c.239_240A | frameshift substitution | 0.36 |
| Case 5 | 5 | 161524661 | 161524661 | GABRG2 | A | AT | NM_198904:c.345_345delinsAT | frameshift substitution | 0.39 |
| Case 5 | 5 | 79366147 | 79366147 | THBS4 | G | A | NM_003248:p.G456R | nonsynonymous SNV | 0.38 |
| Case 5 | 6 | 38645102 | 38645102 | GLO1 | A | C | NM_006708:p.L175W | nonsynonymous SNV | 0.06 |
| Case 5 | 6 | 84630868 | 84630868 | CYB5R4 | A | G | NM_016230:p.K211R | nonsynonymous SNV | 0.39 |
| Case 5 | 7 | 141836369 | 141836369 | LOC93432 | C | A | NM_001293626:p.T259K | nonsynonymous SNV | 0.08 |
| Case 5 | 8 | 86180728 | 86180728 | CA13 | G | C | NM_198584:p.D181H | nonsynonymous SNV | 0.07 |
| Case 5 | 9 | 126776313 | 126776313 | LHX2 | A | G | NM_004789:p.Y65C | nonsynonymous SNV | 0.43 |
| Case 5 | 9 | 138235267 | 138235267 | C9orf62 | C | A | NM_173520:p.T8N | nonsynonymous SNV | 0.43 |
| Case 5 | 10 | 30315095 | 30315095 | KIAA1462 | C | T | NM_020848:p.E1328K | nonsynonymous SNV | 0.41 |
| Case 5 | 11 | 124955027 | 124955027 | SLC37A2 | G | T | NM_198277:p.A414S | nonsynonymous SNV | 0.69 |
| Case 5 | 12 | 58174046 | 58174046 | METTL21B | G | A | NM_206914:p.C146Y | nonsynonymous SNV | 0.43 |
| Case 5 | 14 | 23495328 | 23495333 | PSMB5 | TAGATC | T | NM_001130725:c.757_762A | frameshift substitution | 0.40 |
| Case 5 | 14 | 47426640 | 47426640 | MDGA2 | C | A | NM_182830:p.G378W | nonsynonymous SNV | 0.14 |
| Case 5 | 15 | 26793250 | 26793250 | GABRB3 | G | A | NM_001278631:p.S286L | nonsynonymous SNV | 0.41 |
| Case 5 | 15 | 23889605 | 23889605 | MAGEL2 | G | T | NM_019066:p.Y1095X | stopgain SNV | 0.44 |
| Case 5 | 16 | 69782907 | 69782907 | NOB1 | T | C | NM_014062:p.I214V | nonsynonymous SNV | 0.83 |
| Case 5 | 17 | 27016420 | 27016420 | SUPT6H | G | C | NM_003170:p.E1061D | nonsynonymous SNV | 0.40 |
| Case 5 | 17 | 34870290 | 34870290 | MYO19 | T | C | NM_025109:p.Q240R | nonsynonymous SNV | 0.39 |
| Case 5 | 18 | 50929196 | 50929196 | DCC | G | T | NM_005215:p.R956S | nonsynonymous SNV | 0.45 |
| Case 5 | 19 | 57325615 | 57325615 | PEG3 | C | T | NM_006210:p.V1399M | nonsynonymous SNV | 0.41 |
| Case 5 | 19 | 51015446 | 51015446 | ASPDH | G | C | NM_001114598:p.P252R | nonsynonymous SNV | 0.32 |
| Case 5 | 19 | 58189680 | 58189680 | ZSCAN4 | T | C | NM_152677:p.S237P | nonsynonymous SNV | 0.35 |
| Case 5 | X | 12817326 | 12817326 | PRPS2 | C | G | NM_002765:p.S41R | nonsynonymous SNV | 0.31 |
| Case 5 | X | 18195744 | 18195744 | BEND2 | A | T | NM_153346:p.D525E | nonsynonymous SNV | 0.34 |
| Case 5 | X | 41332990 | 41332990 | NYX | A | G | NM_022567:p.H95R | nonsynonymous SNV | 0.36 |
| Case 5 | X | 70774430 | 70774430 | OGT | G | A | NM_181673:p.R229H | nonsynonymous SNV | 0.30 |
